# Observation-Based Diagnostics of Reactive Nitrogen Recycling through HONO Heterogenous Production: Divergent Implications for Ozone Production and Emission Control

**DOI:** 10.1021/acs.est.3c07967

**Published:** 2024-06-17

**Authors:** Kezhen Chong, Yuhang Wang, Mingming Zheng, Hang Qu, Ruixiong Zhang, Young Ro Lee, Yi Ji, Lewis Gregory Huey, Hua Fang, Wei Song, Zheng Fang, Cheng Liu, Yang Gao, Jianhui Tang, Xinming Wang

**Affiliations:** † School of Earth and Atmospheric Sciences, Georgia Institute of Technology, Atlanta, Georgia 30332, United States; ‡ School of Chemical and Environmental Engineering, Wuhan Polytechnic University, Wuhan 430024, China; § Guangzhou Institute of Geochemistry, Chinese Academy of Sciences, Guangzhou 510640, China; ∥ University of Science and Technology of China, Hefei 230026, China; ⊥ Key Laboratory of Marine Environment and Ecology, Ministry of Education of China, Ocean University of China, Qingdao 266100, China; # Yantai Institute of Coast Zone Research, CAS, Yantai 264003, China

**Keywords:** HONO, photoactive heterogeneous production, ozone production, emission control

## Abstract

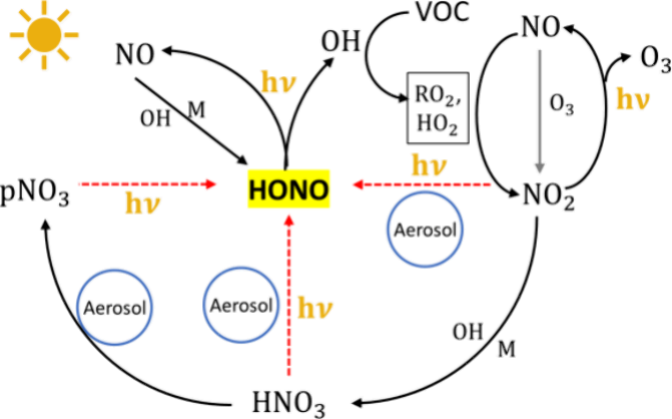

Understanding of nitrous acid (HONO) production is crucial to photochemical studies, especially in polluted environments like eastern China. In-situ measurements of gaseous and particulate compositions were conducted at a rural coastal site during the 2018 spring Ozone Photochemistry and Export from China Experiment (OPECE). This data set was applied to investigate the recycling of reactive nitrogen through daytime heterogeneous HONO production. Although HONO levels increase during agricultural burning, analysis of the observation data does not indicate more efficient HONO production by agricultural burning aerosols than other anthropogenic aerosols. Box and 1-D modeling analyses reveal the intrinsic relationships between nitrogen dioxide (NO_2_), particulate nitrate (pNO_3_), and nitric acid (HNO_3_), resulting in comparable agreement between observed and simulated HONO concentrations with any one of the three heterogeneous HONO production mechanisms, photosensitized NO_2_ conversion on aerosols, photolysis of pNO_3_, and conversion from HNO_3_. This finding underscores the uncertainties in the mechanistic understanding and quantitative parametrizations of daytime heterogeneous HONO production pathways. Furthermore, the implications for reactive nitrogen recycling, ozone (O_3_) production, and O_3_ control strategies vary greatly depending on the HONO production mechanism. On a regional scale, the conversion of HONO from pNO_3_ can drastically enhance O_3_ production, while the conversion from NO_2_ can reduce O_3_ sensitivity to NOx changes in polluted eastern China.

## Abstract 2

Analysis of an extensive observation dataset reveals observation ambiguity in the underlying heterogeneous HONO production mechanism and divergent implications for ozone production and emission control.

## 1 Introduction

Nitrous acid (HONO) is a crucial component in boundary layer photochemistry, playing a significant role in the production of the hydroxyl radical (OH) via fast photolysis.^1−4^ Understanding HONO formation mechanisms advances our knowledge of photochemical processes to better predict and regulate regional air pollution, such as the formation of ozone (O_3_) and secondary organic aerosols.^5−8^

HONO is produced through the gas-phase reaction of nitric oxide (NO) and OH (R1).^9^ Direct emissions from combustion activities, such as vehicle exhaust and biomass burning (BB) can also contribute to HONO levels.^10−15^ In recent years, observations in a variety of locations have found other daytime HONO sources to be important.^16,17^ Soil emissions of HONO have been found to be potentially important for regions with livestock farming or after fertilization, highlighting the need of detailed quantification in future studies.^18^ Moreover, various HONO production pathways have been proposed based on field measurements and laboratory experiments, with heterogeneous reactions highlighted as crucial contributors.^19−22^ For example, studies have demonstrated the importance of heterogeneous nitrogen dioxide (NO_2_) conversion on ground surfaces (eq S3) as an important nocturnal HONO source.^23^ However, this source and gas-phase production still cannot explain observed daytime HONO losses.^24−27^ Aerosol reactions were recognized as a contributor to HONO productions as early as the 1990s.^28^ Research by Ammann et al. detected HONO production from NO_2_ on suspended soot particles, and highlighted the important role of aerosols in HONO production.^29^ Since then, numerous observations have reported HONO production from heterogeneous reactions on aerosols, including photosensitized conversion of NO_2_ (Supporting Information eqs S4–S5),^30−38^ conversion from adsorbed nitric acid (HNO_3_) (Supporting Information eq S9),^39−41^ and photolysis of particulate nitrate (pNO_3_) (Supporting Information eqs S6–S8).^42−45^ More details regarding these HONO sources will be discussed in Section 2.2.
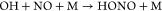
1

Although the precise mechanism of aerosol-induced HONO production is still a subject of debate,^46−48^ heterogeneous mechanisms have been included into atmospheric chemistry simulations and have shown promising agreement with observed levels of HONO. Various HONO budget analyses have been conducted, yet the prevailing mechanism responsible for daytime HONO production beyond R1 remains uncertain.^49−52^ This is due in part to large uncertainties in key reaction parameters of heterogeneous daytime HONO production, such as the reactive uptake coefficient of NO_2_, the enhancement factor of pNO_3_ photolysis rate (jpNO_3_), and the HONO yield coefficient (Y_HONO_) from HNO_3_ conversion, as well as variations in precursor concentrations observed in different campaigns.

Furthermore, environmental factors, such as BB^53−55^ and aerosol acidity,^56,57^ can impact HONO production. This provides additional challenges to characterize HONO sources. For example, Nie et al.^53^ found that the conversion rate of NO_2_ to HONO in BB plumes is twice that in non-BB plumes, suggesting that BB aerosol composition can enhance NO_2_ conversion efficiencies of BB aerosols. Aerosol acidity can affect the volatility of aerosol components and gas-particle partitioning; and HONO heterogeneous production tends to increase with decreasing pH.^58−60^

In this study, we test these proposed HONO heterogeneous mechanisms, including photosensitized NO_2_ conversion on aerosols, photolysis of pNO_3_, and conversion from HNO_3_, using a comprehensive data set of gaseous and particulate compositions measured at a rural coastal site during the 2018 spring Ozone Photochemistry and Export from China Experiment (OPECE).^61^ Observations at a remote location are ideal for diagnosing photochemical processes due in part to generally less-complex emission characteristics compared to urban regions. In this work we investigate the impact of BB and aerosol acidity on observed HONO concentrations. Focusing on the observations not affected by BB, we also analyze the intrinsic photochemical relationships between NO_2_, pNO_3_, and HNO_3_ in the remote boundary layer and how the relationships can obscure the diagnostics of HONO heterogeneous production mechanisms. Lagrangian box model simulations are used to demonstrate the utility of diagnosing O_3_ enhancements by the recycling of reactive nitrogen species through HONO production on aerosols. We also investigate the implications of the different HONO heterogeneous production mechanisms for O_3_ control strategy.

## 2 Materials and Methods

### 2.1 Observations

The OPECE campaign was carried out at the Yellow River Delta Ecology Research Station of the Coastal Wetland in Dongying, Shandong, China. The rural coastal site (37.76°N, 118.98°E) is located in the Yellow River Delta region near the Shandong Yellow River Delta National Nature Reserve.^62^ The closest urban area, Dongying, is located approximately 50 km southwest of the site. From 23 March to 22 April of 2018, surface concentrations of HONO were measured by a Long Path Absorption Photometer (LOPAP).^63−67^ Additionally, measurements of NOx (NO+NO_2_), O_3_, acetonitrile (CH_3_CN), carbon monoxide (CO), volatile organic compounds (VOCs), aerosol size distribution, NO_2_ photolysis rate (jNO_2_), and meteorological parameters, including pressure, temperature, and relative humidity (RH) during this period were obtained. Detailed instrument information is provided in Supporting Information Table S1 and previous studies.^61^

Ambient aerosol surface area (S_A_) was derived using observation data and accounting for the hygroscopic effect,^68^ using the observed RH to estimate the radius ratio of ambient to dry aerosols (Supporting Information Figure S1, eqs S1–S2). Aerosol chemical composition was measured by both an aerosol mass spectrometer (AMS) and PM_2.5_ filter samples. Comparisons of daily inorganic ion concentrations reported by the two instruments demonstrate reasonable agreement with p-values <0.05 and measurement ratios ranging from 0.83 to 1.1 (Supporting Information Figure S2). The AMS instrument samples particles with size <1 μm. Therefore, AMS data are generally lower than the filter measurements. In our study, we combined the two data sets to obtain pNO_3_ and other inorganic aerosol concentrations. Further details regarding data preparation, including the derivations of ambient aerosol surface area, pNO_3_ concentration, and aerosol pH and gas-particulate partitioning calculations, can be found in the Supporting Information Text S1.

### 2.2 Photochemical Models, Source Parameterizations, and Simulation Cases

In this study, we utilize the Regional chEmical trAnsport Model (REAM) in both one- and zero-dimensional configurations^69−71^ to evaluate the recycling of reactive nitrogen species through heterogeneous HONO production and its impacts on O_3_ formation. The model incorporates 30 vertical layers in the troposphere, and the condensed chemistry mechanism from the GEOS-Chem model (v9–02)^72^ for the Ox-NOx-VOCs photochemistry. For VOCs, ≥ 3C alkenes, ≥ 4C alkanes, and high reactivity aromatics are lumped while preserving the OH reactivities. Photolysis rates are calculated based on cloud fraction and optical depths simulated by the Weather Research and Forecasting (WRF) model and subsequently scaled using jNO_2_ observations. To ensure realistic meteorological conditions, observed data are used to constrain meteorological parameters such as temperature, RH, wind velocities and wind directions. Vertical mixing is included using eddy diffusion coefficient (K_*zz*_) simulated by WRF. An averaged diurnal profile of mixing layer height diagnosed by K_*zz*_^73^ is shown in Supporting Information Figure S3. We constrain the model at a 1 min time step using surface observations of O_3_, NO_2_, NO, CO, and selected VOCs. Averaged midmorning to afternoon (10:00–15:00 LT) mixing ratios of VOCs are shown in Supporting Information Figure S4.

To investigate the relationship between HONO production and potential photoactive production pathways, we calculated the missing HONO source strength (pHONO) by
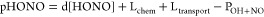
2where, d[HONO] represents the net change of HONO calculated from observations for each 1 min time step, L_chem_ denotes the chemical loss of HONO through photolysis and gas-phase reactions, L_transport_ denotes the loss of HONO through vertical transport, and P_OH+NO_ denotes the production of HONO through the gas-phase reaction of NO and OH. Primary emissions are not considered for this rural coastal site. Since we focus on using pHONO to diagnose daytime HONO sources, the impact of regional transport is not considered either.

Hourly pHONO data are calculated in the baseline 1-D model simulation by constraining surface HONO and other chemical concentrations to the observations (same chemical setup as case B in Table 1) using eq 2. To explain observation-based pHONO, five additional 1-D model simulations were conducted (Table 1). In case S0, HONO production from NO_2_ conversion on ground surface upon dry deposition of NO_2_ was parametrized with a yield coefficient of HONO production (*f*) of 0.24 (Supporting Information eq S3) to reproduce nighttime HONO observations following Liu et al.^24^ As in the previous study, an uptake coefficient of 10^–6^ of NO_2_ uptake to aerosol surfaces under dark conditions was assigned.^74^

**Table 1:** Simulation Cases in This Study

Cases	Configuration
B	NO + OH + M → HONO + M
S0	B + NO_2_ conversions on ground (*f* = 0.24) and on aerosols (γ = 10^–6^) (Supporting Information eq. S3)
S1	S0 + photosensitized NO_2_ conversions on aerosol γ = max (5 × 10^–7^×SWR, 10^–6^) (Supporting Information Eqs. S4–S5)
S2–1	S0 + photolysis of pNO_3_, jpNO_3_= 80 × jHNO_3_ (Supporting Information eqs. S6–S7)
S2–2	S0 + photolysis of pNO_3_, jpNO_3_= EF(pNO_3_, a = 3 × 10^4^)×jHNO_3_ (Supporting Information eqs. S6–S8)
S3	S0 + HONO from HNO_3_, Y_HONO_ = 0.45 (Supporting Information Eq. S9)
F0	Free-running S0
F1	F0 + photosensitized NO_2_ conversions on aerosol γ = max (5 × 10^–7^×SWR, 10^–6^)
F2–1	F0 + photolysis of pNO_3_, jpNO_3_= EF × jHNO_3_, EF = 80
F2–2	F0 + photolysis of pNO_3_, jpNO_3_= EF(pNO_3_, 3 × 10^4^)×jHNO_3_
F3	F0 + HONO from HNO_3_, Y_HONO_ = 0.45

To reproduce daytime HONO observations, three photoactive HONO production mechanisms were implemented in model simulations (Supporting Information eqs S4–S9) on top of S0. The parameters were chosen by considering the values and uncertainties from previous studies and minimizing the simulation errors (root-mean-square error, RMSE) of midmorning to afternoon (10:00–15:00 LT) HONO simulations (see Section 3.2). In S1, we considered a first-order enhancement on aerosol uptake of NO_2_ from short wave radiation (SWR),^24^ in which γ = 5 × 10^–7^ × SWR, to simulate daytime enhancements of pHONO. In S2, photolysis of pNO_3_^42^ was implemented in two subsets. In S2–1, the pNO_3_ photolysis rate (jpNO_3_) was scaled from the photolysis rate of gas-phase HNO_3_ (jHNO_3_) by a constant enhancement factor (EF) of 80.^50^ However, a recent study by Andersen et al.^44^ indicated that EF depends on bulk pNO_3_ concentrations and can be parametrized as a Langmuir function of pNO_3_. In S2–2, EF was computed as a function of the concentration of pNO_3_ (Supporting Information eq S8),^75^ ranging from 20 to 3600 with a median value of 106. Photolysis of nitrate in coarse particles has been found to contribute to HONO production at coastal sites.^76^ In our study, this mechanism is not included due to a lack of coarse particle measurements. This omission may lead to a high bias in the estimated EF. A recent experimental study^77^ has reported HONO production proportional to the production of HNO_3_ (P(HNO_3_)) from photooxidation of NO_2_, and proposed HONO production involving adsorbed HNO_3_. Utilizing this proportional relationship, in S3, we parametrized HONO production with a yield coefficient (Y_HONO_, Supporting Information eq S9) of 0.45. More details of HONO source parametrizations are available in Supporting Information Text S2. The RMSEs of the simulated HONO compared to observations are 0.031, 0.024, 0.019, and 0.019 for S1, S2–1, S2–2, and S3.

Ground sources of HONO have been recognized as potentially important contributors.^25,78^ The HONO isotope observations in China show significant influences of fertilizer applications and livestock farms.^18^ Ground conversion from NO_2_ is found to be a minor HONO source in that study. In our study, the Dongying site is not affected by fertilizer applications or livestock farms. The ground HONO source from NO_2_ is estimated based on nighttime HONO observations. If we assume that the ground source of HONO (in S0) with *f* having a first-order dependence on SWR,^79^ the best fit of the observed HONO gives *f* = min(1, 0.24 + 0.76*SWR/800) since *f* cannot be physically larger than 100%. Under this scenario, *f* reaches 100% during 10:30 to 14:30, but the simulated HONO concentrations still have a low bias compared to the observations (Supporting Information Figure S5). A conversion efficiency of 100% poses stringent requirements for ground surface properties. Equivalent maximum *f* values reported in prior literature^33,80^ are 20% to 40%, much less than 100%; their daily average *f* values are less than or comparable to the nonphotosensitive *f* value used in this study. In terms of simulating observed HONO concentrations and the effect of heterogeneous NO_2_ to HONO conversion on O_3_, the conversion of NO_2_ to HONO on aerosols is equivalent to the conversion on ground surface. Therefore, in this study, the aerosol conversion of NO_2_ to HONO can be thought as representing the combined photosensitized conversions from NO_2_ to HONO on both aerosol and ground surfaces. Similarly, potential photoactive conversions of HONO from HNO_3_ or pNO_3_ deposited on ground surfaces are included in the respective aerosol conversion. During our study period, HNO_3_ predominantly partitions into the particulate phase^25^ (Supporting Information Figure S6) and dry deposition of particulates is slower compared to that of NO_2_. Considering that the dry deposition of HNO_3_ is much faster,^81^ we acknowledge the possible contribution of deposited HNO_3_ on ground to HONO.^82,83^ Since the observations are insufficient to diagnose this contribution and it is implicitly included in the respective aerosol conversion pathway, the effect of this contribution on O_3_ is not separately analyzed in this study.

A major chemical impact of heterogeneous HONO production in S1–S3 is the recycling of reactive nitrogen. To understand the effect of this recycling on PO_3_, we conducted a set of Lagrangian free-running box-model simulations (F0 – F3) to investigate the evolution of plume aging. F0 represents the control case using the same chemical reactions as S0 (case Control). F1 incorporates photosensitized uptake of NO_2_ on aerosol surfaces as in S1. F2–1 considers the photolysis of pNO_3_ with a constant EF as in S2–1 (EF = 80), while F2–2 incorporates a changing EF as in S2–2 (changing EF). Lastly, case F3 includes the production of HONO from HNO_3_ as in S3. The models are initialized with average concentrations of observed O_3_, NO, NO_2_, HONO, CO, and VOCs and started at midnight.

## 3 Results and Discussion

### 3.1 pHONO Dependence on Biomass Burning and Aerosol Acidity

Supporting Information Figure S7 shows the time series of observed O_3_, NOx, HONO, pNO_3_, and S_A_ during the OPECE campaign. The average HONO concentration was 0.39 ± 0.27 parts per billion by volume (ppbv) with a maximum of 1.54 ppbv recorded at the site. These HONO levels have been reported in measurements from other rural coastal sites in China,^84−86^ and some urban/suburban areas in Europe,^17,87^ much higher than those observed in clean coastal regions.^88^ The average concentrations of pNO_3_ and S_A_ were 8 ± 16.8 μg/m^3^ and 527 ± 382 μm^2^/cm^3^, respectively. The diurnal profiles of HONO and other species were also examined (Supporting Information Figure S8). The diurnal profile of HONO illustrates a nighttime accumulation followed by a sharp decrease in the early morning due to rapid photolysis. A slower decrease was observed at noon, indicating the presence of a photoactive daytime HONO source. The average NOx concentration was 13.6 ± 10.4 ppbv, consistent with levels observed at a background site in the North China Plain (NCP) region.^89^ However, notably higher O_3_ levels, exceeding 100 ppbv at times, were observed, with an average peak concentration of 70 ppbv. This elevated O_3_ level underscores the need to gain a better understanding of springtime photochemistry in China. In this study, we focus on investigating the impact of photoactive HONO sources on O_3_ enhancements, as explained in more detail in Section 3.3.

**Figure 1:**
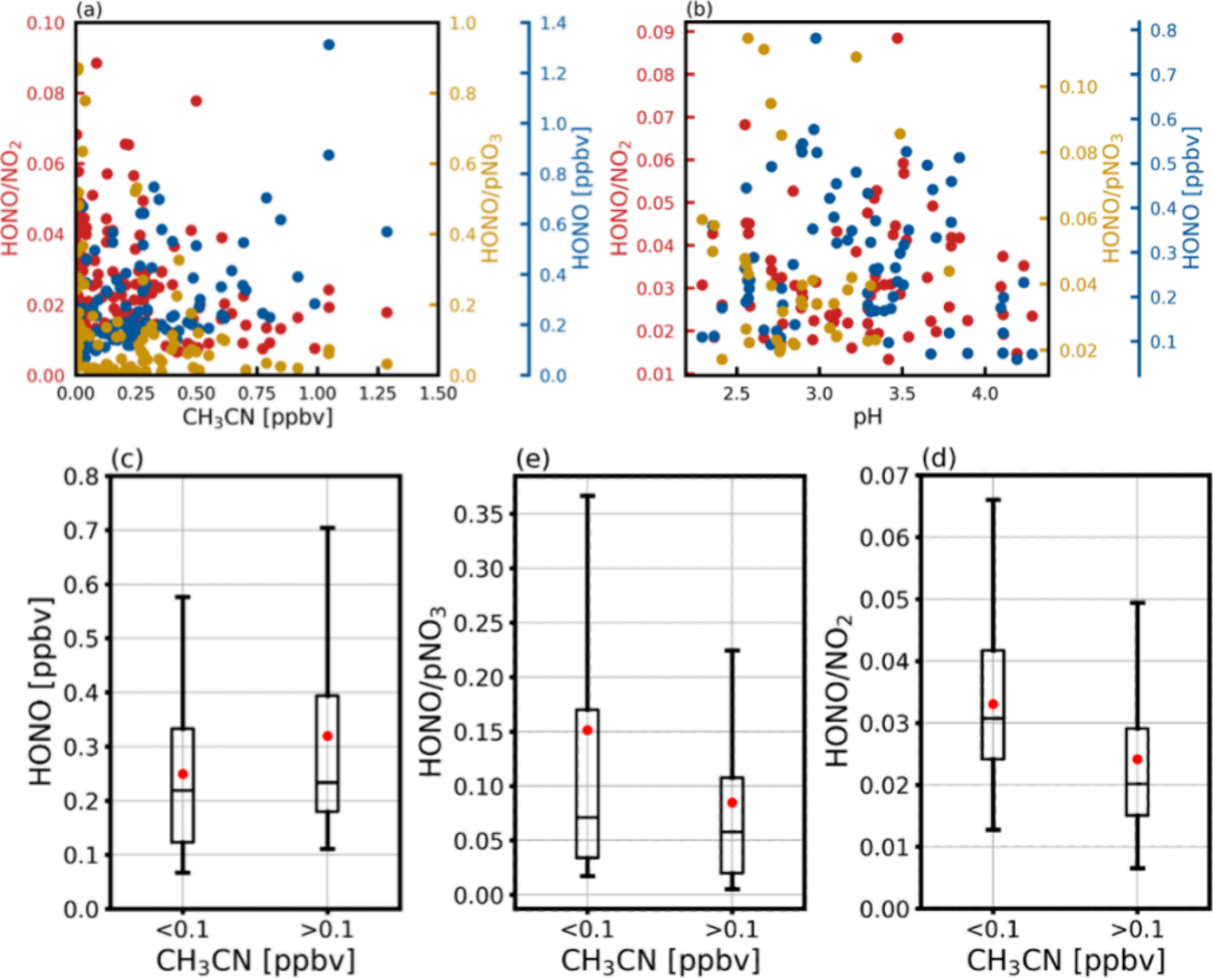
(a) Scatter plots of HONO (blue), HONO/NO_2_ (red), and HONO/pNO_3_ (yellow) as functions of CH_3_CN. (b) Same as (a) but as functions of aerosol pH. (c) Box plots of HONO concentrations for clean (CH_3_CN < 0.1) and BB (CH_3_CN > 0.1) airmasses, where the mean value is denoted by the red dot. And the median is denoted by black line. (d) and (e) are same as (c) but for HONO/NO_2_ and HONO/pNO_3_, respectively.

BB can significantly contribute to regional air pollution, including HONO, through both direct emissions and secondary production. The long lifetime and source-specific characteristics of CH_3_CN make it a widely used tracer for BB.^90−93^ During the measurement campaign, NOx, S_A_, and organic aerosols showed concurrent enhancements with CH_3_CN (Supporting Information Figure S9), indicating the representativeness of CH_3_CN as an indicator of BB impact at this site. For observations with elevated levels of CH_3_CN, we investigated BB impacts on mixing ratios and production pathways of HONO. A previous study reported increased HONO/NO_2_ ratios (2.8% to 6.6%) in BB affected plumes,^53^ suggesting efficient secondary production of HONO on BB aerosols. In our study, we examined the daytime HONO/NO_2_ and HONO/pNO_3_ ratios, which served as indicators for NO_2_ and pNO_3_ conversion efficiencies to HONO, respectively. We analyzed correlations of HONO, HONO/NO_2_, and HONO/pNO_3_ with CH_3_CN (Supporting Information Table S2). Despite the significant positive correlation between HONO and CH_3_CN, consistent with previous studies,^12,13,92,94,95^ we did not find a positive correlation between either ratio with CH_3_CN (Figure 1(a)). To further characterize BB-impacted air masses, we employed a threshold of 0.1 ppbv CH_3_CN to define BB-impacted and clean airmasses.^96,97^ We compared the daytime (8:00–17:00) averaged HONO, HONO/NO_2_, and HONO/pNO_3_ between these two groups. The average HONO concentrations increased from 0.25 to 0.33 ppbv (Figure 1(c)) due to BB. However, the ratios did not show significant differences (Figure 1(d), (e)). We also analyzed data from an agricultural burning event near the site on 31 March 2018^61,62^ and compared the ratios in BB and clean airmasses (Supporting Information Figure S10). While HONO was significantly enhanced during the burning event, the median ratios still showed little difference from those of clean airmasses, except for a slightly higher averaged HONO/NO_2_ ratio than that of the clean airmasses. To minimize the impact of rapid photolysis loss of HONO, nighttime data was also investigated (Supporting Information Figure S11 (a)). A similar pattern emerged, showing a significant positive correlation between HONO and CH_3_CN, insignificant correlations between either HONO/NO_2_ or HONO/pNO_3_ ratio and CH_3_CN. As these ratios may be affected by the enhancements of NOx and pNO_3_ during BB,^98^ it is difficult to conclude whether the increased HONO levels can be attributed to direct emissions or secondary production. Normalized excess mixing ratio (NEMR) has been utilized to quantify fire emissions. Here, we calculated NEMR for HONO relative to NO_2_, i.e., ΔHONO/ΔNO_2_=(HONO^BB^-HONO^nBB^)/(NO_2_^BB^-NO_2_^nBB^), and compared that with previous studies focusing on direct HONO emissions from biomass burning. The NEMR measured in our study ranges from 1.8% for daytime hours (8:00–17:00) to 4.2% during nighttime (20:00–05:00), much lower than the 20% to 140% reported in previous studies.^95^ This result suggests that the effect of direct agricultural burning emissions of HONO at the OPECE site is much less than those found for forest fires in previous studies.

In addition to BB, previous studies show that heterogeneous HONO production is affected by aerosol acidity through altering the partitioning between nitrite (NO_2_^–^) and HONO.^99^ Here, we investigated the correlation between pH and heterogeneous HONO production using non-BB data. No statistically significant correlations are found during daytime (Figure 1(b), Supporting Information Table S3). At night (Supporting Information Figure S11(b), Table S3), HONO shows a weak negative correlation with aerosol pH. The negative correlation between pH and S_A_ reflects in part higher sulfate concentrations in large particles in China.^100^ This contributes to the negative correlation of HONO/NO_2_ with pH, as higher S_A_ leads to more conversion of NO_2_ to HONO. The positive correlation between HONO/pNO_3_ and pH reflects in part the degassing of pNO_3_ at higher acidity.^101^

### 3.2 Recycling of Reactive Nitrogen through Photoactive Heterogeneous HONO Production

Here we address the question to see if a comprehensive in situ observation data set can be applied to test the validity of the proposed heterogeneous mechanisms, including photosensitized NO_2_ conversion on aerosol, photolysis of pNO_3_ and HONO yield from adsorbed HNO_3_. A caveat in this analysis is that NO_2_, HNO_3_, and pNO_3_ are all part of the reactive nitrogen family and they have dependent relationships, which are reflected in the correlation analysis. To avoid the impact of BB, we focus on the observations not affected by BB, which comprises 55% of the total available data (Supporting Information Figure S12).

**Figure 2:**
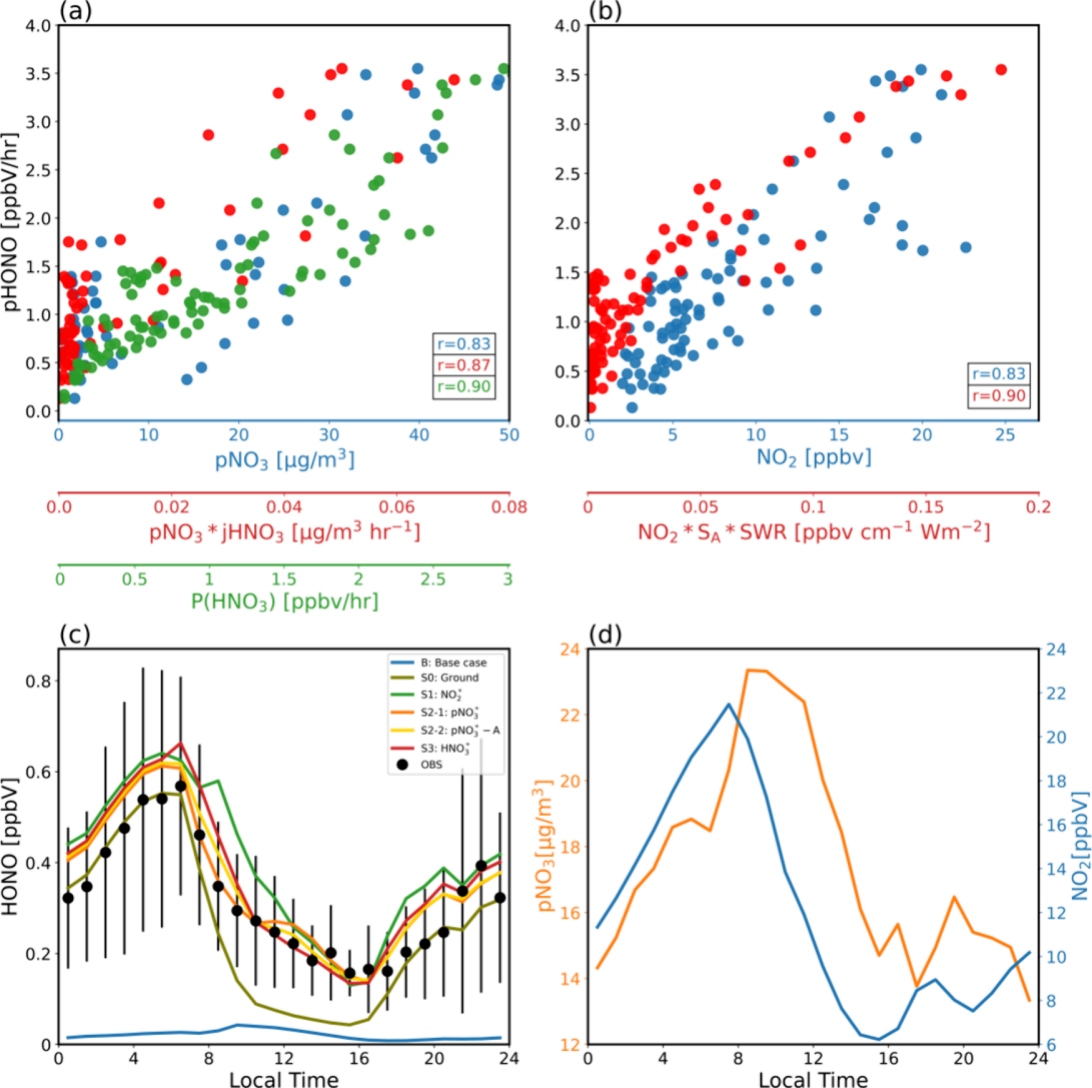
(a) Scatter plot of pHONO as functions of pNO_3_ (blue), pNO_3_ × jHNO_3_ (red), and P(HNO_3_) (green). The corresponding correlation coefficients are shown at the bottom right corner of the plot. (b) Scatter plot of pHONO as functions of NO_2_ (blue) and NO_2_ × S_A_ × SWR (red), and the corresponding correlation coefficients. (c) Observed diurnal HONO (black dots) with standard deviation (black vertical lines) and simulated HONO under the six cases (B, S0–S3) as described in Table 1. (d) Mean diurnal profiles of pNO_3_ (orange) and NO_2_ (blue) during the measurement period. All panels show the non-BB data.

We assess each production pathway by examining the correlations of pHONO with the corresponding production terms associated with each pathway. A strong positive correlation lends observational support to the existence of the source, whereas a lack of correlation can be considered observational evidence for excluding the production pathway as a major contributor. Figure 2(a) shows a significant positive correlation between pHONO and pNO_3_, with a correlation coefficient r = 0.83. This correlation strengthens to r = 0.87 when considering the case of pNO_3_ × jHNO_3_ (Figure 2(a)). Additionally, although the parametrization for the production pathway via HNO_3_ is based on a smog experiment, which requires further validation and investigation, a strong correlation between pHONO and P(HNO_3_) is found with r = 0.9 in our study. We also find a significant correlation between pHONO and the production terms of photosensitized NO_2_ conversion on aerosols, with r increasing from 0.82 between pHONO and NO_2_ to 0.90 for that with NO_2_ × S_A_ × SWR (Figure 2(b)). Correlation statistics can be found in Supporting Information Table S4. These strong correlations reflect the intrinsic relationships among the precursors of heterogeneous HONO production.^102,103^

To further assess the photoactive HONO production mechanisms, we conducted a set of constrained simulations (S0–S3, Table 1) incorporating additional HONO sources as described in Section 2. Consistent with previous studies, the observed HONO levels consistently exceed those predicted by R1 (Figure 2(c): B) throughout the day. Nocturnal HONO levels are explained by heterogeneous NO_2_ conversion on the ground surface with f = 0.24, while daytime HONO concentrations remain underestimated (Figure 2(c): S0).

After incorporating photoactive HONO production mechanisms (S1–S3), the model with any one of the mechanisms can reproduce the observed HONO levels within one standard deviation (Figure 2(c): S1–S3). A slightly higher bias of S1 in the morning hours (8:00–10:00) is seen, this is due to the fact that a constant scaling factor of 5 × 10^–7^ is used for γ, minimizing the 10:00–15:00 RMSE yields a higher bias in the morning when both NO_2_ and S_A_ reach peak values. Importantly, the derived key parameters for HONO production, obtained by minimizing the simulation errors (i.e., scaling factor for γ (5 × 10^–4^), EF (80 or 106), and Y_HONO_ (0.45)), are comparable to those reported in previous experimental and modeling studies: [2 × 10^–5^, 1 × 10^–3^], [8, 700], and 0.53, respectively (Supporting Information Table S5). This agreement underscores the difficulty of assessing the underlying HONO heterogeneous production mechanism due to the intrinsic relationships among reactive nitrogen species. For example, higher HONO concentrations can promote pNO_3_ production,^25^ and the photolysis of pNO_3_ can recycle reactive nitrogen from a reservoir species back into more reactive gaseous species, thereby sustaining atmospheric NO_2_ levels.^104−106^ Moreover, as atmospheric HNO_3_ is produced through the reaction of OH and NO_2_, the production rate of HONO via this process is directly proportional to NO_2_. Given these intertwined relationships, it becomes challenging to establish a definitive causal relationship between HONO production and the proposed mechanisms solely through photochemical analysis of in situ observations.

However, these mechanisms have very different implications for the recycling of reactive nitrogen. The conversion from NO_2_ to HONO has the lowest impact since both species are short-lived. In springtime, the gas-particulate partitioning of nitrate strongly favors the formation of pNO_3_ from gaseous HNO_3_.^25^ Therefore, the conversion of HNO_3_ to HONO reduces the lifetime of HNO_3_ and the formation of pNO_3_, effectively enhancing the recycling of reactive nitrogen. The photolysis of pNO_3_ significantly reduces the lifetime of the most long-lived reactive nitrogen reservoir and makes the entire inorganic reactive nitrogen family photochemically active, speeding up the recycling of reactive nitrogen the most.

We acknowledge that some reaction parameters derived in this study may appear larger than the values observed in laboratory experiments. For instance, the uptake coefficient for NO_2_ of 5 × 10^–4^ is greater than typical experimental results (Supporting Information Table S5). Two key points should be highlighted. First, the parameters were derived under the assumption that the unexplained daytime HONO is exclusively produced through the targeted mechanism. In the real atmosphere, multiple mechanisms may contribute to HONO simultaneously. Consequently, the parameters obtained in this study represent upper limits. Second, our primary focus is on understanding the implications for reactive nitrogen recycling and, consequently, O_3_ production. We are not aiming to establish the optimal kinetics parameters or identify the dominant mechanism, which cannot be accomplished through photochemical modeling of field observations as shown in our analysis. In the following section, we delve deeper into exploring the impact of HONO heterogeneous production on O_3_ production. It will become evident that, even if all these mechanisms yield the same amount of HONO, their effects on O_3_ production differ significantly due to varying efficiencies in reactive nitrogen recycling.

### 3.3 Sensitivity of O_3_ Production to the Heterogeneous Reactive Nitrogen Mechanism

The different implications of the heterogeneous HONO production mechanisms for the recycling of reactive nitrogen also strongly affect the production of O_3_. In this section, we investigate the changes of O_3_ chemistry by heterogeneous HONO production. The chemical analysis is isolated from meteorological impacts. As such, the box model simulations assume that the composition of an air mass is isolated from mixing and other meteorological processes. This analysis provides qualitative insights into the vastly different impacts of different heterogeneous HONO production pathways on O_3_ production.

**Figure 3:**
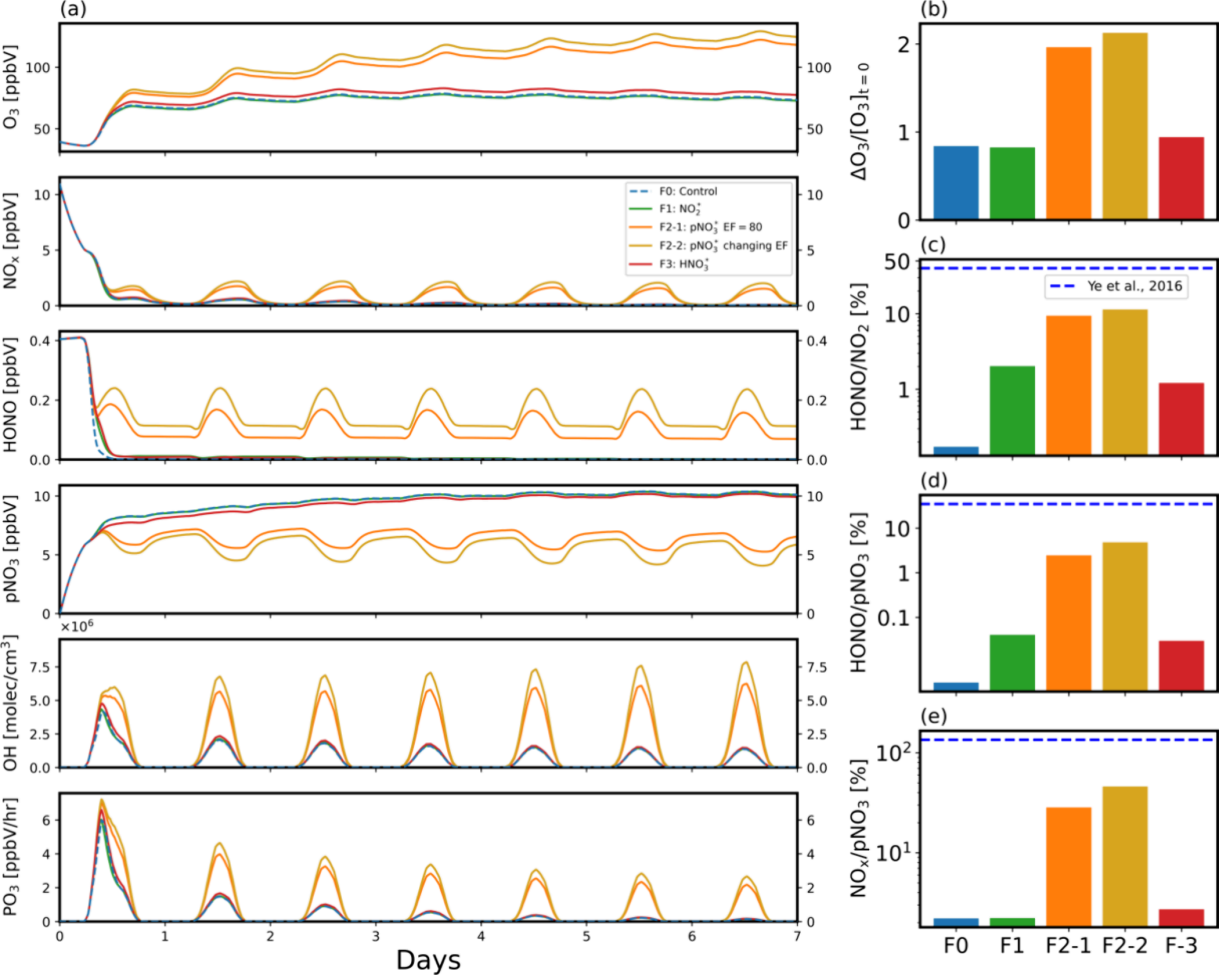
(a) Simulated evolutions of O_3_, NOx, HONO, pNO_3_, OH, and PO_3_ in a Lagrangian box model for 7 days under cases F0 – F3. (b) O_3_ enhancement ratios, defined as the relative O_3_ increase from the initial O_3_ concentration for cases F0 – F3. (c), (d), and (e) are simulated HONO/NO_2_, HONO/pNO_3_ and NOx/pNO_3_ ratios. The dashed lines denote measured HONO/NO_2_, HONO/pNO_3_ and NOx/pNO_3_ values from Ye et al.^104^

We use Lagrangian box model simulations to analyze the chemical evolutions of isolated airmasses and investigate the consequences of the heterogeneous HONO production mechanisms (Table 1: F0 – F3). Figure 3(a) shows the divergent evolutions of O_3_, NOx, OH, and O_3_ production rate (PO_3_) among the HONO production mechanisms. Compared to the control case (F0) without HONO production from aerosols, the addition of photoactive conversion of NO_2_ (F1) or adsorbed HNO_3_ to HONO (F3) has a transient impact on reactive nitrogen, OH, and O_3_. In contrast, the impact from F2 cases via pNO_3_ photolysis is much larger and longer lasting. The difference lies in how the heterogeneous HONO production mechanism affects the recycling between NOx and pNO_3_. The conversion of NO_2_ to HONO on aerosols partitions reactive nitrogen into HONO, which effectively increases HONO/NO_2_ and HONO/pNO_3_ when compared to F0 (Figure 3(c), (d)), but does not significantly affect the ratio of NOx/pNO_3_ (Figure 3(e)). In the case of F3, the addition of HONO source reduces the production of HNO_3_ and hence the concentration of pNO_3_ by converting it to HONO and then to NOx, resulting in an increased NOx/pNO_3_ ratio (Figure 3(e)),^107^ in addition to enhancing HONO/NO_2_ and HONO/pNO_3_ similar to F1. The photolysis of HONO adds a larger radical source and can speed up O_3_ production.^108^ However, one O_3_ is lost for each conversion of NO_2_ to HONO. In contrast, the conversion from HNO_3_ to HONO does not incur this O_3_ loss and therefore produces more O_3_ than F1 (Figure 3(b)). These effects are most pronounced in the first day but diminish over time as NOx is converted to pNO_3_, which serves as a permanent sink for reactive nitrogen in these two cases.

In cases F2–1 and F2–2, however, the conversion of pNO_3_ to HONO allows a full recycling of inorganic reactive nitrogen species,^106,109−111^ thus the highest NOx/pNO_3_ ratio (Figure 3(e)). In some previous modeling studies, in addition to HONO, NO_2_ is also included as a product of pNO_3_ photolysis, this could lead to direct recycling from pNO_3_ to NOx, thus increasing NOx/pNO_3_ ratio and subsequent O_3_ production even more. Daytime HONO and NOx concentrations are sustained at significant levels, leading to high OH concentrations and O_3_ production and a tripling of O_3_ concentrations of >100 ppbv after a week (Figure 3(a)). The increase of O_3_ is twice as much in F2–1 and F2–2 as that of F0, F1 or F3 (Figure 3(b)). The difference between F2–1 and F2–2 is smaller compared to their differences from F1 and F3. A recent study^112^ implementing HONO conversion from NO_2_ and pNO_3_ into a chemistry-climate model found a reduction effect on O_3_ driven by HONO conversion from NO_2_ and a strong enhancement of NOx caused by HONO conversion from pNO_3_. A necessary condition to simulate an O_3_ reduction is that NO from HONO photolysis can only produce a fractional (<1) O_3_ despite the additional OH from HONO photolysis. This could occur when OH reacts with O_3_ due to a lack of reactive VOCs. Figure 3 shows that it is not the case in a polluted boundary layer.

Ye et al.^104^ reported rapid cycling of pNO_3_ to HONO from aircraft measurements over the North Atlantic Ocean in the marine boundary layer, based on observed high HONO production with low-level NOx present. We reexamined their observation data using the simulation results from OPECE. Following the observation-model comparison procedure by Ye et al., we used model outputs at 2:30 pm on the second diurnal cycle, which represents a 1.5-day airmass transport from the coastal site in the marine boundary layer, to be compared to their observations. The observed HOHO/NO_2_, HONO/pNO_3_, and NOx/pNO_3_ ratios reported by Ye et al. are much higher than our simulation results (Figure 3 (c) - (e)), although our observations during OPECE are more comparable to the observed values reported by other ground-based studies (Supporting Information Table S6). Therefore, the marine observations by Ye et al. appear to suggest a chemical environment of extremely fast recycling of reactive nitrogen not seen over polluted land areas.^88,113^

Figure 3(b) shows that O_3_ production rate can be much higher from the pNO_3_ conversion mechanism (F2–1 and F2–2) than the two other mechanisms (F1 and F3) in comparison to the case of no heterogeneous HONO production (F0). However, the effects depend on the amount of reactive nitrogen. To understand how the relationship between O_3_ and NOx responds to these different mechanisms,^114,115^ we conducted box model simulations for 3 h under noontime conditions with initial NOx mixing ratios in the range of 1–25 ppbv. Similar results are obtained when longer integration hours are used, but the differences of F2–1 and F2–2 from the other cases increase with time.

**Figure 4:**
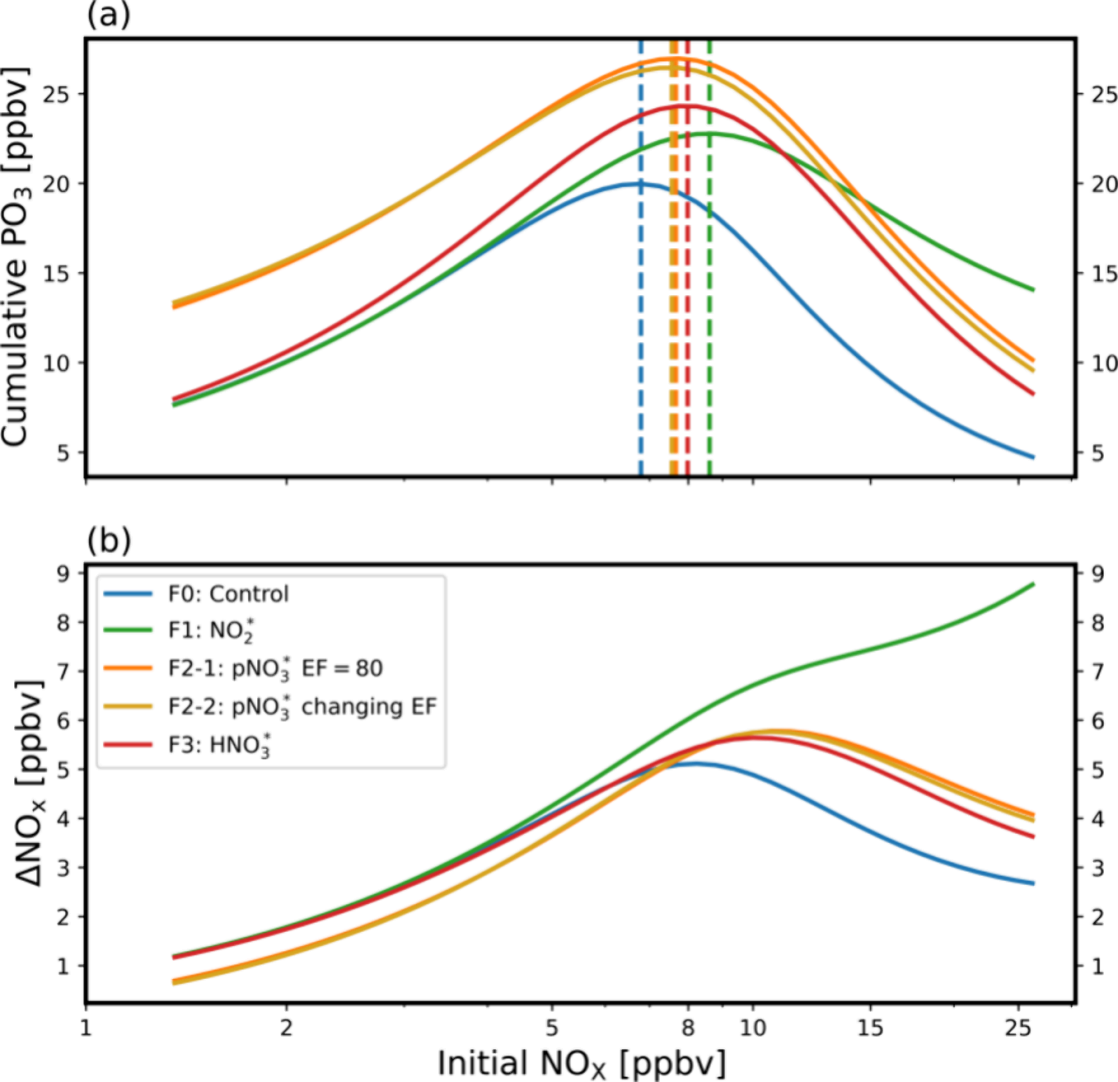
(a) Cumulative PO_3_ as a function of initial NOx for cases F0 - F3. The dashed lines denote NOx levels at which cumulative PO_3_ reaches its peak values. (b) Loss of NOx (ΔNOx) as a function of initial NOx.

A few features emerge from the modeling analysis. Figure 4(a) shows the cumulative PO_3_ first increases with NOx and then starts to decrease with increasing NOx. The NOx concentration at which the cumulative PO_3_ maximizes is the transition from NOx-limited to VOC-limited O_3_ production.^71,108^ The critical initial NOx concentration for this transition increases when heterogeneous HONO production is introduced (Figure 4). The largest increase occurs with the NO_2_ conversion mechanism (F1) because the OH production from HONO photolysis speeds up the conversion of NOx to its reservoir, HNO_3_. In F2 and F3 cases, HNO_3_ can be effectively recycled back to NOx but not in F1. Consequently, the reduction of NOx is fastest in F1 due to HONO production. In a polluted urban environment, where O_3_ production decreases as NOx increases, this faster NOx reduction and increased OH production tends to increase PO_3_. Previous modeling studies have also shown elevated O_3_ levels via F1 in high-NOx regions.^116^ As the urban plume ages and is transported regionally to low-NOx areas, however, this mechanism could lead to significant NOx reductions.^112^ It is therefore expected that pNO_3_ recycling (F2) will increase PO_3_ much more than NO_2_ recycling (F1) on a regional scale. Supporting Information Figure S13 shows the ozone production efficiency (OPE), defined as the cumulative PO_3_ in a three-hour period (ΔO_3_) per NOx consumed (ΔNOx) as a function of initial NOx concentration. The NO_2_ conversion case (F1) is slightly higher than the case without heterogeneous HONO production (F0) under high-NOx conditions. The pNO_3_ conversion case (F2–1 and F2–2) increases OPE by a factor of 2–4 compared to F0. The HNO_3_ conversion case (F3) is more similar to (and higher than) the NO_2_ conversion case (F1) under low NOx conditions and approaches the pNO_3_ conversion cases (F2–1 and F2–2) under high NOx conditions.

### 3.4 Implications

Observed HONO levels are enhanced with increasing aerosol acidity and agricultural burning. However, analysis of the observation data does not indicate more efficient HONO production by agricultural burning aerosols. The NEMR of HONO over NO_2_ for agricultural burning in our study ranges from 1.8% in daytime to 4.2% at night, which is much lower than those reported for forest fires. Aerosol acidity is not found to enhance the non-BB aerosol HONO production efficiency in daytime data. The analysis of nighttime data is inconclusive since other factors may also lead to the observed correlations of aerosol acidity with HONO/NO_2_ and HONO/pNO_3_ ratios at night.

Modeling analysis of the OPECE gaseous and particulate measurements shows clear evidence for daytime heterogeneous production of HONO on aerosols in agreement with previous studies. By incorporating either photosensitized NO_2_ conversion on aerosols, photolysis of pNO_3_, or conversion from HNO_3_, the model can reproduce the observed HONO concentrations when other reactive nitrogen and chemical species are constrained by the observations. This modeling equivalency reflects the intrinsic relationships between NO_2_, pNO_3_, and HNO_3_ in the chemical system, indicating that inland in situ observations may not provide sufficient constraints to investigate the underlying mechanism of heterogeneous HONO production. This finding highlights the uncertainties in both the mechanistic understanding and quantitative parametrizations of daytime heterogeneous HONO production pathways. It appears necessary to conduct Lagrangian experiments away from sources such as in the marine boundary layer since the three mechanisms have drastically different predictions of HONO/NO_2_, HONO/pNO_3_, and NOx/pNO_3_ ratios (Figure 3(c)-(e)) after 1–2 days of chemical aging. Being able to follow the evolutions of these ratios can provide more information on the underlying mechanism.

The maritime observations by Ye et al.^104^ are not as ideal as a Lagrangian experiment but can be carried out more easily. Their observations suggest a chemical environment with much faster reactive nitrogen recycling than the one found in OPECE or other inland data sets, indicating that additional processes may contribute to efficient reactive nitrogen recycling through HONO production over the ocean. However, it also implies much higher O_3_ production in offshore regions than in current model predictions. A survey of OPECE observations did not provide evidence for high O_3_ airmass transport from offshore regions, which may be related to prevailing westerly conditions during OPECE.

Quantifying the mechanism of heterogeneous HONO production on aerosols has profound implications for understanding O_3_ pollution and is important to formulating effective mitigation strategies on a regional scale. The conversion of HONO from pNO_3_ can drastically increase the lifetime of NOx and the cumulative O_3_ production during aging (Figures 3 and 4) compared to the other two mechanisms. It has the potential to substantially elevate O_3_ concentrations in regions located downwind from emission sources and to amplify O_3_ levels even in distant marine environments, potentially exerting a global influence.

On the other hand, the conversion of HONO from NO_2_ on aerosols can move the transition regime, when O_3_ production becomes insensitive to NOx, into higher NOx conditions and significantly flatten the PO_3_ decrease as NOx increases under high-NOx conditions (Figure 4(a)). The faster removal of NOx (Figure 4(b)) also implies a lower effect of background O_3_ production in downwind regions compared to the other cases since the OPEs of case F1 are among the lowest under low NOx conditions (Supporting Information Figure S13). The net effect is to reduce O_3_ sensitivity to NOx changes. It may help explain the observations that O_3_ concentrations in China have not changed much over the past decade (Supporting Information Figure S14) despite a ∼ 50% reduction in NOx (Supporting Information Figure S15)^117−120^

Despite the increasingly recognized significance of HONO over the years, the underlying production mechanisms of HONO are not understood and have considerable uncertainties. The kinetics parameters for heterogeneous HONO production have large uncertainties, and future laboratory studies are needed for improving the accuracy of these parameters. This study focuses on understanding their impacts on O_3_ production on a regional scale. Previous modeling studies have incorporated various HONO sources to address the underestimation of daytime HONO and to understand their corresponding impacts on photochemical pollutants. However, the simulated impacts on reactive nitrogen recycling and O_3_ are tied to the parametrizations of these sources in the models, which can introduce large uncertainties. Hence caution is warranted in the incoproration of HONO sources into models, as different production pathways can have vastly different implications, as found in this study.

## Supporting Information Available

Details on the instrument used, preparation of observational data, and parametrizations of HONO sources; summary of the observed pollutant concentrations; detailed statistical test results for analyses on biomass burning impact, aerosol acidity impact, and HONO source evaluations; comparisons with previous studies on key kinetic parameters, and HONO/NO_2_/pNO_3_ measurements; O_3_ production efficiency with changing NO_2_ concentration; observed O_3_ and NO_2_ levels in eastern China from 2014 to 2022 (PDF)

## References

[ref1] Dusanter S.; Vimal D.; Stevens P. S.; Volkamer R.; Molina L. T.; Baker A.; Meinardi S.; Blake D.; Sheehy P.; Merten A.; Zhang R.; Zheng J.; Fortner E. C.; Junkermann W.; Dubey M.; Rahn T.; Eichinger B.; Lewandowski P.; Prueger J.; Holder H. Measurements of OH and HO2 concentrations during the MCMA-2006 field campaign – Part 2: Model comparison and radical budget. Atmos. Chem. Phys. 2009, 9 (18), 6655–6675. 10.5194/acp-9-6655-2009.

[ref2] Kleffmann J. Daytime formation of nitrous acid: A major source of OH radicals in a forest. Geophys. Res. Lett. 2005, 32 (5), L05818 10.1029/2005GL022524.

[ref3] Kim S.; VandenBoer T. C.; Young C. J.; Riedel T. P.; Thornton J. A.; Swarthout B.; Sive B.; Lerner B.; Gilman J. B.; Warneke C.; Roberts J. M.; Guenther A.; Wagner N. L.; Dubé W. P.; Williams E.; Brown S. S. The primary and recycling sources of OH during the NACHTT-2011 campaign: HONO as an important OH primary source in the wintertime. Journal of Geophysical Research: Atmospheres 2014, 119 (11), 6886–6896. 10.1002/2013JD019784.

[ref4] Alicke B.; Platt U.; Stutz J. Impact of nitrous acid photolysis on the total hydroxyl radical budget during the Limitation of Oxidant Production/Pianura Padana Produzione di Ozono study in Milan. Journal of Geophysical Research: Atmospheres 2002, 107 (D22), LOP 9-1–LOP 9-17. 10.1029/2000JD000075.

[ref5] Lu X.; Wang Y.; Li J.; Shen L.; Fung J. C. H. Evidence of heterogeneous HONO formation from aerosols and the regional photochemical impact of this HONO source. Environmental Research Letters 2018, 13 (11), 114002 10.1088/1748-9326/aae492.

[ref6] Xing L.; Wu J.; Elser M.; Tong S.; Liu S.; Li X.; Liu L.; Cao J.; Zhou J.; El-Haddad I.; Huang R.; Ge M.; Tie X.; Prévôt A. S. H.; Li G. Wintertime secondary organic aerosol formation in Beijing–Tianjin–Hebei (BTH): contributions of HONO sources and heterogeneous reactions. Atmospheric Chemistry and Physics 2019, 19 (4), 2343–2359. 10.5194/acp-19-2343-2019.

[ref7] Liu P.; Xue C.; Ye C.; Liu C.; Zhang C.; Wang J.; Zhang Y.; Liu J.; Mu Y. The Lack of HONO Measurement May Affect the Accurate Diagnosis of Ozone Production Sensitivity. ACS Environmental Au 2023, 3 (1), 18–23. 10.1021/acsenvironau.2c00048. 37101842 PMC10125324

[ref8] Li Q.; Zhang L.; Wang T.; Wang Z.; Fu X.; Zhang Q. ″New″ Reactive Nitrogen Chemistry Reshapes the Relationship of Ozone to Its Precursors. Environ. Sci. Technol. 2018, 52 (5), 2810–2818. 10.1021/acs.est.7b05771. 29406712

[ref9] Pagsberg P.; Bjergbakke E.; Ratajczak E.; Sillesen A. Kinetics of the gas phase reaction OH + NO(+ M) HONO(+ M) and the determination of the UV absorption cross sections of HONO. Chem. Phys. Lett. 1997, 272, 383 10.1016/S0009-2614(97)00576-9.

[ref10] Burling I. R.; Yokelson R. J.; Griffith D. W. T.; Johnson T. J.; Veres P.; Roberts J. M.; Warneke C.; Urbanski S. P.; Reardon J.; Weise D. R.; Hao W. M.; de Gouw J. Laboratory measurements of trace gas emissions from biomass burning of fuel types from the southeastern and southwestern United States. Atmospheric Chemistry and Physics 2010, 10 (22), 11115–11130. 10.5194/acp-10-11115-2010.

[ref11] Veres P.; Roberts J. M.; Burling I. R.; Warneke C.; de Gouw J.; Yokelson R. J. Measurements of gas-phase inorganic and organic acids from biomass fires by negative-ion proton-transfer chemical-ionization mass spectrometry. Journal of Geophysical Research: Atmospheres 2010, 115 (D23), D23302 10.1029/2010JD014033.

[ref12] Neuman J. A.; Trainer M.; Brown S. S.; Min K. E.; Nowak J. B.; Parrish D. D.; Peischl J.; Pollack I. B.; Roberts J. M.; Ryerson T. B.; Veres P. R. HONO emission and production determined from airborne measurements over the Southeast U.S. Journal of Geophysical Research: Atmospheres 2016, 121 (15), 9237–9250. 10.1002/2016JD025197.

[ref13] Theys N.; Volkamer R.; Müller J. F.; Zarzana K. J.; Kille N.; Clarisse L.; De Smedt I.; Lerot C.; Finkenzeller H.; Hendrick F.; Koenig T. K.; Lee C. F.; Knote C.; Yu H.; Van Roozendael M. Global nitrous acid emissions and levels of regional oxidants enhanced by wildfires. Nature Geoscience 2020, 13 (10), 681–686. 10.1038/s41561-020-0637-7.

[ref14] Roberts J. M.; Stockwell C. E.; Yokelson R. J.; de Gouw J.; Liu Y.; Selimovic V.; Koss A. R.; Sekimoto K.; Coggon M. M.; Yuan B.; Zarzana K. J.; Brown S. S.; Santin C.; Doerr S. H.; Warneke C. The nitrogen budget of laboratory-simulated western US wildfires during the FIREX 2016 Fire Lab study. Atmos. Chem. Phys. 2020, 20 (14), 8807–8826. 10.5194/acp-20-8807-2020.

[ref15] Xu L.; Crounse J. D.; Vasquez K. T.; Allen H.; Wennberg P. O.; Bourgeois I.; Brown S. S.; Campuzano-Jost P.; Coggon M. M.; Crawford J. H.; DiGangi J. P.; Diskin G. S.; Fried A.; Gargulinski E. M.; Gilman J. B.; Gkatzelis G. I.; Guo H.; Hair J. W.; Hall S. R.; Halliday H. A.; Hanisco T. F.; Hannun R. A.; Holmes C. D.; Huey L. G.; Jimenez J. L.; Lamplugh A.; Lee Y. R.; Liao J.; Lindaas J.; Neuman J. A.; Nowak J. B.; Peischl J.; Peterson D. A.; Piel F.; Richter D.; Rickly P. S.; Robinson M. A.; Rollins A. W.; Ryerson T. B.; Sekimoto K.; Selimovic V.; Shingler T.; Soja A. J.; St. Clair J. M.; Tanner D. J.; Ullmann K.; Veres P. R.; Walega J.; Warneke C.; Washenfelder R. A.; Weibring P.; Wisthaler A.; Wolfe G. M.; Womack C. C.; Yokelson R. J. Ozone chemistry in western U.S. wildfire plumes. Science Advances 2021, 7 (50), eabl3648 10.1126/sciadv.abl3648. 34878847 PMC8654285

[ref16] Li X.; Rohrer F.; Hofzumahaus A.; Brauers T.; Häseler R.; Bohn B.; Broch S.; Fuchs H.; Gomm S.; Holland F.; Jäger J.; Kaiser J.; Keutsch F. N.; Lohse I.; Lu K.; Tillmann R.; Wegener R.; Wolfe G. M.; Mentel T. F.; Kiendler-Scharr A.; Wahner A. Missing Gas-Phase Source of HONO Inferred from Zeppelin Measurements in the Troposphere. Science 2014, 344 (6181), 292–296. 10.1126/science.1248999. 24744373

[ref17] Michoud V.; Colomb A.; Borbon A.; Miet K.; Beekmann M.; Camredon M.; Aumont B.; Perrier S.; Zapf P.; Siour G.; Ait-Helal W.; Afif C.; Kukui A.; Furger M.; Dupont J. C.; Haeffelin M.; Doussin J. F. Study of the unknown HONO daytime source at a European suburban site during the MEGAPOLI summer and winter field campaigns. Atmos. Chem. Phys. 2014, 14 (6), 2805–2822. 10.5194/acp-14-2805-2014.

[ref18] Zhang Q.; Liu P.; Wang Y.; George C.; Chen T.; Ma S.; Ren Y.; Mu Y.; Song M.; Herrmann H.; Mellouki A.; Chen J.; Yue Y.; Zhao X.; Wang S.; Zeng Y. Unveiling the underestimated direct emissions of nitrous acid (HONO). Proc. Natl. Acad. Sci. U. S. A. 2023, 120 (35), e2302048120 10.1073/pnas.2302048120. 37603738 PMC10468620

[ref19] Lammel G.; Cape J. N. Nitrous acid and nitrite in the atmosphere. Chem. Soc. Rev. 1996, 25 (5), 361–369. 10.1039/cs9962500361.

[ref20] Finlayson-Pitts B. J.; Wingen L. M.; Sumner A. L.; Syomin D.; Ramazan K. A. The heterogeneous hydrolysis of NO2 in laboratory systems and in outdoor and indoor atmospheres: An integrated mechanism. Phys. Chem. Chem. Phys. 2003, 5 (2), 223–242. 10.1039/b208564j.

[ref21] Kleffmann J.; Becker K.; Wiesen P. Heterogeneous NO2 conversion processes on acid surfaces: possible atmospheric implications. Atmos. Environ. 1998, 32 (16), 2721–2729. 10.1016/S1352-2310(98)00065-X.

[ref22] Reisinger A. R. Observations of HNO2 in the polluted winter atmosphere: possible heterogeneous production on aerosols. Atmos. Environ. 2000, 34 (23), 3865–3874. 10.1016/S1352-2310(00)00179-5.

[ref23] Tuite K.; Thomas J. L.; Veres P. R.; Roberts J. M.; Stevens P. S.; Griffith S. M.; Dusanter S.; Flynn J. H.; Ahmed S.; Emmons L.; Kim S.-W.; Washenfelder R.; Young C.; Tsai C.; Pikelnaya O.; Stutz J. Quantifying Nitrous Acid Formation Mechanisms Using Measured Vertical Profiles During the CalNex 2010 Campaign and 1D Column Modeling. Journal of Geophysical Research: Atmospheres 2021, 126 (13), e2021JD034689 10.1029/2021JD034689.

[ref24] Liu Z.; Wang Y.; Costabile F.; Amoroso A.; Zhao C.; Huey L. G.; Stickel R.; Liao J.; Zhu T. Evidence of aerosols as a media for rapid daytime HONO production over China. Environ. Sci. Technol. 2014, 48 (24), 14386–14391. 10.1021/es504163z. 25401515

[ref25] Xue C.; Zhang C.; Ye C.; Liu P.; Catoire V.; Krysztofiak G.; Chen H.; Ren Y.; Zhao X.; Wang J.; Zhang F.; Zhang C.; Zhang J.; An J.; Wang T.; Chen J.; Kleffmann J.; Mellouki A.; Mu Y. HONO Budget and Its Role in Nitrate Formation in the Rural North China Plain. Environ. Sci. Technol. 2020, 54 (18), 11048–11057. 10.1021/acs.est.0c01832. 32808764

[ref26] Zheng J.; Shi X.; Ma Y.; Ren X.; Jabbour H.; Diao Y.; Wang W.; Ge Y.; Zhang Y.; Zhu W. Contribution of nitrous acid to the atmospheric oxidation capacity in an industrial zone in the Yangtze River Delta region of China. Atmospheric Chemistry and Physics 2020, 20 (9), 5457–5475. 10.5194/acp-20-5457-2020.

[ref27] Stutz J.; Alicke B.; Neftel A. Nitrous acid formation in the urban atmosphere: Gradient measurements of NO2 and HONO over grass in Milan, Italy. Journal of Geophysical Research: Atmospheres 2002, 107 (D22), LOP 5-1–LOP 5-15. 10.1029/2001JD000390.

[ref28] Notholt J.; Hjorth J.; Raes F. Formation of HNO2 on aerosol surfaces during foggy periods in the presence of NO and NO2. Atmospheric Environment. Part A. General Topics 1992, 26 (2), 211–217. 10.1016/0960-1686(92)90302-2.

[ref29] Ammann M.; Kalberer M.; Jost D. T.; Tobler L.; Rossler E.; Piguet D.; W G. H.; Baltensperger U. Heterogeneous production of nitrous acid on soot in polluted air masses. Nature 1998, 395, 157 10.1038/25965.

[ref30] George C.; Strekowski R. S.; Kleffmann J.; Stemmler K.; Ammann M. Photoenhanced uptake of gaseous NO2 on solid organic compounds: a photochemical source of HONO?. Faraday Discuss. 2005, 130, 195–210. 10.1039/b417888m. 16161785

[ref31] Ramazan K. A.; Syomin D.; Finlayson-Pitts B. J. The photochemical production of HONO during the heterogeneous hydrolysis of NO2. Phys. Chem. Chem. Phys. 2004, 6 (14), 3836–3843. 10.1039/b402195a.

[ref32] Gustafsson R. J.; Orlov A.; Griffiths P. T.; Cox R. A.; Lambert R. M. Reduction of NO2 to nitrous acid on illuminated titanium dioxide aerosol surfaces: implications for photocatalysis and atmospheric chemistry. Chem. Commun. 2006, (37), 3936–3938. 10.1039/b609005b. 17268676

[ref33] Stemmler K.; Ammann M.; Donders C.; Kleffmann J.; George C. Photosensitized reduction of nitrogen dioxide on humic acid as a source of nitrous acid. Nature 2006, 440 (7081), 195–198. 10.1038/nature04603. 16525469

[ref34] Ndour M.; D’Anna B.; George C.; Ka O.; Balkanski Y.; Kleffmann J.; Stemmler K.; Ammann M. Photoenhanced uptake of NO2 on mineral dust: Laboratory experiments and model simulations. Geophys. Res. Lett. 2008, 35 (5), L05812 10.1029/2007GL032006.

[ref35] Wang S.; Ackermann R.; Spicer C. W.; Fast J. D.; Schmeling M.; Stutz J. Atmospheric observations of enhanced NO2-HONO conversion on mineral dust particles. Geophys. Res. Lett. 2003, 30 (11), 1595 10.1029/2003GL017014.

[ref36] Jiang Y.; Xue L.; Gu R.; Jia M.; Zhang Y.; Wen L.; Zheng P.; Chen T.; Li H.; Shan Y.; Zhao Y.; Guo Z.; Bi Y.; Liu H.; Ding A.; Zhang Q.; Wang W. Sources of nitrous acid (HONO) in the upper boundary layer and lower free troposphere of North China Plain: insights from the Mount Tai Observatory. Atmos. Chem. Phys. 2020, 20, 12115 10.5194/acp-20-12115-2020.

[ref37] Colussi A. J.; Enami S.; Yabushita A.; Hoffmann M. R.; Liu W. G.; Mishra H.; Goddard W. A. 3rd. Tropospheric aerosol as a reactive intermediate. Faraday Discuss. 2013, 165, 407–420. 10.1039/c3fd00040k. 24601015

[ref38] Ricker H. M.; Leonardi A.; Navea J. G. Reduction and Photoreduction of NO2 in Humic Acid Films as a Source of HONO, ClNO, N2O, NOX, and Organic Nitrogen. ACS Earth and Space Chemistry 2022, 6 (12), 3066–3077. 10.1021/acsearthspacechem.2c00282. 36561196 PMC9762234

[ref39] Jiang Y.; Hoffmann E. H.; Tilgner A.; Aiyuk M. B. E.; Andersen S. T.; Wen L.; van Pinxteren M.; Shen H.; Xue L.; Wang W.; Herrmann H. Insights Into NOx and HONO Chemistry in the Tropical Marine Boundary Layer at Cape Verde During the MarParCloud Campaign. Journal of Geophysical Research: Atmospheres 2023, 128 (16), e2023JD038865 10.1029/2023JD038865.

[ref40] Tsai C.; Spolaor M.; Colosimo S. F.; Pikelnaya O.; Cheung R.; Williams E.; Gilman J. B.; Lerner B. M.; Zamora R. J.; Warneke C.; Roberts J. M.; Ahmadov R.; de Gouw J.; Bates T.; Quinn P. K.; Stutz J. Nitrous acid formation in a snow-free wintertime polluted rural area. Atmos. Chem. Phys. 2018, 18 (3), 1977–1996. 10.5194/acp-18-1977-2018.

[ref41] Laufs S.; Kleffmann J. Investigations on HONO formation from photolysis of adsorbed HNO3 on quartz glass surfaces. Phys. Chem. Chem. Phys. 2016, 18 (14), 9616–9625. 10.1039/C6CP00436A. 26997156

[ref42] Ye C.; Zhang N.; Gao H.; Zhou X. Photolysis of Particulate Nitrate as a Source of HONO and NOx. Environ. Sci. Technol. 2017, 51 (12), 6849–6856. 10.1021/acs.est.7b00387. 28505434

[ref43] Zhou X.; Zhang N.; TerAvest M.; Tang D.; Hou J.; Bertman S.; Alaghmand M.; Shepson P. B.; Carroll M. A.; Griffith S.; Dusanter S.; Stevens P. S. Nitric acid photolysis on forest canopy surface as a source for tropospheric nitrous acid. Nature Geoscience 2011, 4 (7), 440–443. 10.1038/ngeo1164.

[ref44] Andersen S. T.; Carpenter L. J.; Reed C.; Lee J. D.; Chance R.; Sherwen T.; Vaughan A. R.; Stewart J.; Edwards P. M.; Bloss W. J.; Sommariva R.; Crilley L. R.; Nott G. J.; Neves L.; Read K.; Heard D. E.; Seakins P. W.; Whalley L. K.; Boustead G. A.; Fleming L. T.; Stone D.; Fomba K. W. Extensive field evidence for the release of HONO from the photolysis of nitrate aerosols. Science Advances 2023, 9 (3), eadd6266 10.1126/sciadv.add6266. 36652523 PMC9848427

[ref45] Ye C.; Zhou X.; Pu D.; Stutz J.; Festa J.; Spolaor M.; Tsai C.; Cantrell C.; Mauldin Iii R. L.; Weinheimer A.; Hornbrook R. S.; Apel E. C.; Guenther A.; Kaser L.; Yuan B.; Karl T.; Haggerty J.; Hall S.; Ullmann K.; Smith J.; Ortega J. Tropospheric HONO distribution and chemistry in the southeastern US. Atmos. Chem. Phys. 2018, 18 (12), 9107–9120. 10.5194/acp-18-9107-2018.

[ref46] Chai J.; Dibb J. E.; Anderson B. E.; Bekker C.; Blum D. E.; Heim E.; Jordan C. E.; Joyce E. E.; Kaspari J. H.; Munro H.; Walters W. W.; Hastings M. G. Isotopic constraints on wildfire derived HONO. Atmospheric Chemistry and Physics Discussion 2021, 10.5194/acp-2021-225.

[ref47] Romer P. S.; Wooldridge P. J.; Crounse J. D.; Kim M. J.; Wennberg P. O.; Dibb J. E.; Scheuer E.; Blake D. R.; Meinardi S.; Brosius A. L.; Thames A. B.; Miller D. O.; Brune W. H.; Hall S. R.; Ryerson T. B.; Cohen R. C. Constraints on Aerosol Nitrate Photolysis as a Potential Source of HONO and NO x. Environ. Sci. Technol. 2018, 52 (23), 13738–13746. 10.1021/acs.est.8b03861. 30407797

[ref48] Pusede S. E.; VandenBoer T. C.; Murphy J. G.; Markovic M. Z.; Young C. J.; Veres P. R.; Roberts J. M.; Washenfelder R. A.; Brown S. S.; Ren X.; Tsai C.; Stutz J.; Brune W. H.; Browne E. C.; Wooldridge P. J.; Graham A. R.; Weber R.; Goldstein A. H.; Dusanter S.; Griffith S. M.; Stevens P. S.; Lefer B. L.; Cohen R. C. An Atmospheric Constraint on the NO2 Dependence of Daytime Near-Surface Nitrous Acid (HONO). Environ. Sci. Technol. 2015, 49 (21), 12774–12781. 10.1021/acs.est.5b02511. 26436410

[ref49] Yang Y.; Li X.; Zu K.; Lian C.; Chen S.; Dong H.; Feng M.; Liu H.; Liu J.; Lu K.; Lu S.; Ma X.; Song D.; Wang W.; Yang S.; Yang X.; Yu X.; Zhu Y.; Zeng L.; Tan Q.; Zhang Y. Elucidating the effect of HONO on O3 pollution by a case study in southwest China. Sci. Total Environ. 2021, 756, 144127 10.1016/j.scitotenv.2020.144127. 33288267

[ref50] Liu Y.; Lu K.; Li X.; Dong H.; Tan Z.; Wang H.; Zou Q.; Wu Y.; Zeng L.; Hu M.; Min K. E.; Kecorius S.; Wiedensohler A.; Zhang Y. A Comprehensive Model Test of the HONO Sources Constrained to Field Measurements at Rural North China Plain. Environ. Sci. Technol. 2019, 53 (7), 3517–3525. 10.1021/acs.est.8b06367. 30811937

[ref51] Ge Y.; Shi X.; Ma Y.; Zhang W.; Ren X.; Zheng J.; Zhang Y. Seasonality of nitrous acid near an industry zone in the Yangtze River Delta region of China: Formation mechanisms and contribution to the atmospheric oxidation capacity. Atmos. Environ. 2021, 254, 118420 10.1016/j.atmosenv.2021.118420.

[ref52] Shi X.; Ge Y.; Zheng J.; Ma Y.; Ren X.; Zhang Y. Budget of nitrous acid and its impacts on atmospheric oxidative capacity at an urban site in the central Yangtze River Delta region of China. Atmos. Environ. 2020, 238, 117725 10.1016/j.atmosenv.2020.117725.

[ref53] Nie W.; Ding A. J.; Xie Y. N.; Xu Z.; Mao H.; Kerminen V. M.; Zheng L. F.; Qi X. M.; Huang X.; Yang X. Q.; Sun J. N.; Herrmann E.; Petäjä T.; Kulmala M.; Fu C. B. Influence of biomass burning plumes on HONO chemistry in eastern China. Atmospheric Chemistry and Physics 2015, 15 (3), 1147–1159. 10.5194/acp-15-1147-2015.

[ref54] Peng Q.; Palm B. B.; Fredrickson C. D.; Lee B. H.; Hall S. R.; Ullmann K.; Weinheimer A. J.; Levin E.; DeMott P.; Garofalo L. A.; Pothier M. A.; Farmer D. K.; Fischer E. V.; Thornton J. A. Direct Constraints on Secondary HONO Production in Aged Wildfire Smoke From Airborne Measurements Over the Western US. Geophys. Res. Lett. 2022, 49 (15), e2022GL098704 10.1029/2022GL098704.

[ref55] Chai J.; Miller D. J.; Scheuer E.; Dibb J.; Selimovic V.; Yokelson R.; Zarzana K. J.; Brown S. S.; Koss A. R.; Warneke C.; Hastings M. Isotopic characterization of nitrogen oxides (NOx), nitrous acid (HONO), and nitrate (pNO3−) from laboratory biomass burning during FIREX. Atmos. Meas. Technol. 2019, 12 (12), 6303–6317. 10.5194/amt-12-6303-2019.

[ref56] Scharko N. K.; Berke A. E.; Raff J. D. Release of nitrous acid and nitrogen dioxide from nitrate photolysis in acidic aqueous solutions. Environ. Sci. Technol. 2014, 48 (20), 11991–12001. 10.1021/es503088x. 25271384

[ref57] Shi X.; Nenes A.; Xiao Z.; Song S.; Yu H.; Shi G.; Zhao Q.; Chen K.; Feng Y.; Russell A. G. High-Resolution Data Sets Unravel the Effects of Sources and Meteorological Conditions on Nitrate and Its Gas-Particle Partitioning. Environ. Sci. Technol. 2019, 53 (6), 3048–3057. 10.1021/acs.est.8b06524. 30793889

[ref58] Park J. Y.; Lee Y. N. Solubility and decomposition kinetics of nitrous acid in aqueous solution. J. Phys. Chem. 1988, 92 (22), 6294–6302. 10.1021/j100333a025.

[ref59] Mora Garcia S. L.; Pandit S.; Navea J. G.; Grassian V. H. Nitrous Acid (HONO) Formation from the Irradiation of Aqueous Nitrate Solutions in the Presence of Marine Chromophoric Dissolved Organic Matter: Comparison to Other Organic Photosensitizers. ACS Earth and Space Chemistry 2021, 5 (11), 3056–3064. 10.1021/acsearthspacechem.1c00292.

[ref60] VandenBoer T. C.; Markovic M. Z.; Sanders J. E.; Ren X.; Pusede S. E.; Browne E. C.; Cohen R. C.; Zhang L.; Thomas J.; Brune W. H.; Murphy J. G. Evidence for a nitrous acid (HONO) reservoir at the ground surface in Bakersfield, CA, during CalNex 2010. Journal of Geophysical Research: Atmospheres 2014, 119 (14), 9093–9106. 10.1002/2013JD020971.

[ref61] Lee Y.; Huey L. G.; Wang Y.; Qu H.; Zhang R.; Ji Y.; Tanner D. J.; Wang X.; Tang J.; Song W.; Hu W.; Zhang Y. Photochemistry of Volatile Organic Compounds in the Yellow River Delta, China: Formation of O3 and Peroxyacyl Nitrates. Journal of Geophysical Research: Atmospheres 2021, 126 (23), e2021JD035296 10.1029/2021JD035296.

[ref62] Chong K.; Wang Y.; Liu C.; Gao Y.; Boersma K. F.; Tang J.; Wang X. Remote Sensing Measurements at a Rural Site in China: Implications for Satellite NO2 and HCHO Measurement Uncertainty and Emissions From Fires. Journal of Geophysical Research: Atmospheres 2024, 129 (2), e2023JD039310 10.1029/2023JD039310.

[ref63] Kleffmann J.; Heland J.; Kurtenbach R.; Lörzer J. C.; Wiesen P. A new instrument (LOPAP) for the detection of nitrous acid (HONO). Environ. Sci. Pollut. Res. 2002, 9, 48–54.

[ref64] Heland J.; Kleffmann J.; Kurtenbach R.; Wiesen P. A New Instrument To Measure Gaseous Nitrous Acid (HONO) in the Atmosphere. Environ. Sci. Technol. 2001, 35 (15), 3207–3212. 10.1021/es000303t. 11506004

[ref65] Kleffmann J.; Lörzer J. C.; Wiesen P.; Kern C.; Trick S.; Volkamer R.; Rodenas M.; Wirtz K. Intercomparison of the DOAS and LOPAP techniques for the detection of nitrous acid (HONO). Atmos. Environ. 2006, 40 (20), 3640–3652. 10.1016/j.atmosenv.2006.03.027.

[ref66] Reed C.; Brumby C. A.; Crilley L. R.; Kramer L. J.; Bloss W. J.; Seakins P. W.; Lee J. D.; Carpenter L. J. HONO measurement by differential photolysis. Atmos. Meas. Technol. 2016, 9 (6), 2483–2495. 10.5194/amt-9-2483-2016.

[ref67] Crilley L. R.; Kramer L. J.; Ouyang B.; Duan J.; Zhang W.; Tong S.; Ge M.; Tang K.; Qin M.; Xie P.; Shaw M. D.; Lewis A. C.; Mehra A.; Bannan T. J.; Worrall S. D.; Priestley M.; Bacak A.; Coe H.; Allan J.; Percival C. J.; Popoola O. A. M.; Jones R. L.; Bloss W. J. Intercomparison of nitrous acid (HONO) measurement techniques in a megacity (Beijing). Atmospheric Measurement Techniques 2019, 12 (12), 6449–6463. 10.5194/amt-12-6449-2019.

[ref68] Lewis E. R. An examination of Köhler theory resulting in an accurate expression for the equilibrium radius ratio of a hygroscopic aerosol particle valid up to and including relative humidity 100%. Journal of Geophysical Research 2008, 113 (D3), D03205 10.1029/2007JD008590.

[ref69] Liu Z.; Wang Y.; Gu D.; Zhao C.; HUEY L. G.; STICKEL R.; LIAO J.; Shao M.; Zhu T.; Zeng L.; Liu S.-C.; CHANG C.-C.; AMOROSO A.; COSTABILE F. Evidence of Reactive Aromatics As a Major Source of Peroxy Acetyl Nitrate over China. Environ. Sci. Technol. 2010, 44, 7017 10.1021/es1007966. 20707413

[ref70] Zhang Y.; Wang Y.; Chen G.; Smeltzer C.; Crawford J.; Olson J.; Szykman J.; Weinheimer A. J.; Knapp D. J.; Montzka D. D.; Wisthaler A.; Mikoviny T.; Fried A.; Diskin G. Large vertical gradient of reactive nitrogen oxides in the boundary layer: Modeling analysis of DISCOVER-AQ 2011 observations. Journal of Geophysical Research: Atmospheres 2016, 121 (4), 1922–1934. 10.1002/2015JD024203.

[ref71] Qu H.; Wang Y.; Zhang R.; Liu X.; Huey L. G.; Sjostedt S.; Zeng L.; Lu K.; Wu Y.; Shao M.; Hu M.; Tan Z.; Fuchs H.; Broch S.; Wahner A.; Zhu T.; Zhang Y. Chemical Production of Oxygenated Volatile Organic Compounds Strongly Enhances Boundary-Layer Oxidation Chemistry and Ozone Production. Environ. Sci. Technol. 2021, 55 (20), 13718–13727. 10.1021/acs.est.1c04489. 34623137

[ref72] Wang Y.; Logan J. A.; Jacob D. J. Global simulation of tropospheric O3-NOx-hydrocarbon chemistry: 2. Model evaluation and global ozone budget. Journal of Geophysical Research: Atmospheres 1998, 103 (D9), 10727–10755. 10.1029/98JD00157.

[ref73] Li J.; Wang Y.; Zhang R.; Smeltzer C.; Weinheimer A.; Herman J.; Boersma K. F.; Celarier E. A.; Long R. W.; Szykman J. J.; Delgado R.; Thompson A. M.; Knepp T. N.; Lamsal L. N.; Janz S. J.; Kowalewski M. G.; Liu X.; Nowlan C. R. Comprehensive evaluations of diurnal NO2 measurements during DISCOVER-AQ 2011: effects of resolution-dependent representation of NOx emissions. Atmos. Chem. Phys. 2021, 21 (14), 11133–11160. 10.5194/acp-21-11133-2021. 35949546 PMC9359208

[ref74] Kleffmann J.; Becker K. H.; Wiesen P. Heterogeneous NO2 conversion processes on acid surfaces: possible atmospheric implications. Atmos. Environ. 1998, 32 (16), 2721–2729. 10.1016/S1352-2310(98)00065-X.

[ref75] Wang Y.; Wang J.; Wang Y.; Zhang Y.; Woodward-Massey R.; Zhang C.; Kuang Y.; Zhu J.; Shang J.; Li X.; Zeng L.; Lin W.; Ye C. Experimental and kinetic model evaluation of HONO production from surface nitrate photolysis. Atmos. Environ. 2023, 296, 119568 10.1016/j.atmosenv.2022.119568.

[ref76] Tang M.-X.; He L.-Y.; Xia S.-Y.; Jiang Z.; He D.-Y.; Guo S.; Hu R.-Z.; Zeng H.; Huang X.-F. Coarse particles compensate for missing daytime sources of nitrous acid and enhance atmospheric oxidation capacity in a coastal atmosphere. Science of The Total Environment 2024, 915, 170037 10.1016/j.scitotenv.2024.170037. 38232856

[ref77] Song M.; Zhao X.; Liu P.; Mu J.; He G.; Zhang C.; Tong S.; Xue C.; Zhao X.; Ge M.; Mu Y. Atmospheric NOx oxidation as major sources for nitrous acid (HONO). npj Climate and Atmospheric Science 2023, 6 (1), 30 10.1038/s41612-023-00357-8.

[ref78] Stemmler K.; Ndour M.; Elshorbany Y.; Kleffmann J.; D’Anna B.; George C.; Bohn B.; Ammann M. Light induced conversion of nitrogen dioxide into nitrous acid on submicron humic acid aerosol. Atmos. Chem. Phys. 2007, 7 (16), 4237–4248. 10.5194/acp-7-4237-2007.

[ref79] Zhang J.; Lian C.; Wang W.; Ge M.; Guo Y.; Ran H.; Zhang Y.; Zheng F.; Fan X.; Yan C.; Daellenbach K. R.; Liu Y.; Kulmala M.; An J. Amplified role of potential HONO sources in O3 formation in North China Plain during autumn haze aggravating processes. Atmos. Chem. Phys. 2022, 22 (5), 3275–3302. 10.5194/acp-22-3275-2022.

[ref80] Han C.; Yang W.; Wu Q.; Yang H.; Xue X. Heterogeneous Photochemical Conversion of NO2 to HONO on the Humic Acid Surface under Simulated Sunlight. Environ. Sci. Technol. 2016, 50 (10), 5017–5023. 10.1021/acs.est.5b05101. 27074517

[ref81] Williams R. M. A model for the dry deposition of particles to natural water surfaces. Atmospheric Environment (1967) 1982, 16 (8), 1933–1938. 10.1016/0004-6981(82)90464-4.

[ref82] Li X.; Brauers T.; Häseler R.; Bohn B.; Fuchs H.; Hofzumahaus A.; Holland F.; Lou S.; Lu K. D.; Rohrer F.; Hu M.; Zeng L. M.; Zhang Y. H.; Garland R. M.; Su H.; Nowak A.; Wiedensohler A.; Takegawa N.; Shao M.; Wahner A. Exploring the atmospheric chemistry of nitrous acid (HONO) at a rural site in Southern China. Atmospheric Chemistry and Physics 2012, 12 (3), 1497–1513. 10.5194/acp-12-1497-2012.

[ref83] Zhou X.; Huang G.; Civerolo K.; Roychowdhury U.; Demerjian K. L. Summertime observations of HONO, HCHO, and O3 at the summit of Whiteface Mountain, New York. Journal of Geophysical Research: Atmospheres 2007, 112 (D8), D08311 10.1029/2006JD007256.

[ref84] Gu R.; Zheng P.; Chen T.; Dong C.; Wang Y. n; Liu Y.; Liu Y.; Luo Y.; Han G.; Wang X.; Zhou X.; Wang T.; Wang W.; Xue L. Atmospheric nitrous acid (HONO) at a rural coastal site in North China: Seasonal variations and effects of biomass burning. Atmos. Environ. 2020, 229, 117429 10.1016/j.atmosenv.2020.117429.

[ref85] Yang J.; Shen H.; Guo M.-Z.; Zhao M.; Jiang Y.; Chen T.; Liu Y.; Li H.; Zhu Y.; Meng H.; Wang W.; Xue L. Strong marine-derived nitrous acid (HONO) production observed in the coastal atmosphere of northern China. Atmos. Environ. 2021, 244, 117948 10.1016/j.atmosenv.2020.117948.

[ref86] Cui L.; Li R.; Fu H.; Li Q.; Zhang L.; George C.; Chen J. Formation features of nitrous acid in the offshore area of the East China Sea. Sci. Total Environ. 2019, 682, 138–150. 10.1016/j.scitotenv.2019.05.004. 31112815

[ref87] Lee J. D.; Whalley L. K.; Heard D. E.; Stone D.; Dunmore R. E.; Hamilton J. F.; Young D. E.; Allan J. D.; Laufs S.; Kleffmann J. Detailed budget analysis of HONO in central London reveals a missing daytime source. Atmospheric Chemistry and Physics 2016, 16 (5), 2747–2764. 10.5194/acp-16-2747-2016.

[ref88] Zha Q.; Xue L.; Wang T.; Xu Z.; Yeung C.; Louie P. K. K.; Luk C. W. Y. Large conversion rates of NO2 to HNO2 observed in air masses from the South China Sea: Evidence of strong production at sea surface?. Geophys. Res. Lett. 2014, 41 (21), 7710–7715. 10.1002/2014GL061429.

[ref89] Liu X.; Ran L.; Lin W.; Xu X.; Ma Z.; Dong F.; He D.; Zhou L.; Shi Q.; Wang Y. Measurement report: Variations in surface SO2 and NOx mixing ratios from 2004 to 2016 at a background site in the North China Plain. Atmos. Chem. Phys. 2022, 22 (10), 7071–7085. 10.5194/acp-22-7071-2022.

[ref90] Chen J.; Li C.; Ristovski Z.; Milic A.; Gu Y.; Islam M. S.; Wang S.; Hao J.; Zhang H.; He C.; Guo H.; Fu H.; Miljevic B.; Morawska L.; Thai P.; Lam Y. F.; Pereira G.; Ding A.; Huang X.; Dumka U. C. A review of biomass burning: Emissions and impacts on air quality, health and climate in China. Sci. Total Environ. 2017, 579, 1000–1034. 10.1016/j.scitotenv.2016.11.025. 27908624

[ref91] Yuan B.; Liu Y.; Shao M.; Lu S.; Streets D. G. Biomass Burning Contributions to Ambient VOCs Species at a Receptor Site in the Pearl River Delta (PRD), China. Environ. Sci. Technol. 2010, 44 (12), 4577–4582. 10.1021/es1003389. 20507061

[ref92] Juncosa Calahorrano J. F.; Lindaas J.; O’Dell K.; Palm B. B.; Peng Q.; Flocke F.; Pollack I. B.; Garofalo L. A.; Farmer D. K.; Pierce J. R.; Collett J. L.; Weinheimer A.; Campos T.; Hornbrook R. S.; Hall S. R.; Ullmann K.; Pothier M. A.; Apel E. C.; Permar W.; Hu L.; Hills A. J.; Montzka D.; Tyndall G.; Thornton J. A.; Fischer E. V. Daytime Oxidized Reactive Nitrogen Partitioning in Western U.S. Wildfire Smoke Plumes. Journal of Geophysical Research: Atmospheres 2021, 126 (4), e2020JD033484 10.1029/2020JD033484.

[ref93] Hughes D. D.; Christiansen M. B.; Milani A.; Vermeuel M. P.; Novak G. A.; Alwe H. D.; Dickens A. F.; Pierce R. B.; Millet D. B.; Bertram T. H.; Stanier C. O.; Stone E. A. PM2.5 chemistry, organosulfates, and secondary organic aerosol during the 2017 Lake Michigan Ozone Study. Atmos. Environ. 2021, 244, 117939 10.1016/j.atmosenv.2020.117939.

[ref94] Lindaas J.; Pollack I. B.; Garofalo L. A.; Pothier M. A.; Farmer D. K.; Kreidenweis S. M.; Campos T. L.; Flocke F.; Weinheimer A. J.; Montzka D. D.; Tyndall G. S.; Palm B. B.; Peng Q.; Thornton J. A.; Permar W.; Wielgasz C.; Hu L.; Ottmar R. D.; Restaino J. C.; Hudak A. T.; Ku I. T.; Zhou Y.; Sive B. C.; Sullivan A.; Collett J. L.; Fischer E. V. Emissions of Reactive Nitrogen From Western U.S. Wildfires During Summer 2018. Journal of Geophysical Research: Atmospheres 2021, 126 (2), e2020JD032657 10.1029/2020JD032657.

[ref95] Peng Q.; Palm B. B.; Melander K. E.; Lee B. H.; Hall S. R.; Ullmann K.; Campos T.; Weinheimer A. J.; Apel E. C.; Hornbrook R. S.; Hills A. J.; Montzka D. D.; Flocke F.; Hu L.; Permar W.; Wielgasz C.; Lindaas J.; Pollack I. B.; Fischer E. V.; Bertram T. H.; Thornton J. A. HONO Emissions from Western U.S. Wildfires Provide Dominant Radical Source in Fresh Wildfire Smoke. Environ. Sci. Technol. 2020, 54, 5954 10.1021/acs.est.0c00126. 32294377

[ref96] Xu R.; Li X.; Dong H.; Wu Z.; Chen S.; Xin F.; Gao J.; Guo S.; Hu M.; Li D.; Liu Y.; Liu Y.; Lou S.; Lu K.; Meng X.; Wang H.; Zeng L.; Zong T.; Hu J.; Zhang Y. Measurement of gaseous and particulate formaldehyde in the Yangtze River Delta, China. Atmos. Environ. 2020, 224, 117114 10.1016/j.atmosenv.2019.117114.

[ref97] Brock C. A.; Cozic J.; Bahreini R.; Froyd K. D.; Middlebrook A. M.; McComiskey A.; Brioude J.; Cooper O. R.; Stohl A.; Aikin K. C.; de Gouw J. A.; Fahey D. W.; Ferrare R. A.; Gao R. S.; Gore W.; Holloway J. S.; Hübler G.; Jefferson A.; Lack D. A.; Lance S.; Moore R. H.; Murphy D. M.; Nenes A.; Novelli P. C.; Nowak J. B.; Ogren J. A.; Peischl J.; Pierce R. B.; Pilewskie P.; Quinn P. K.; Ryerson T. B.; Schmidt K. S.; Schwarz J. P.; Sodemann H.; Spackman J. R.; Stark H.; Thomson D. S.; Thornberry T.; Veres P.; Watts L. A.; Warneke C.; Wollny A. G. Characteristics, sources, and transport of aerosols measured in spring 2008 during the aerosol, radiation, and cloud processes affecting Arctic Climate (ARCPAC) Project. Atmospheric Chemistry and Physics 2011, 11 (6), 2423–2453. 10.5194/acp-11-2423-2011.

[ref98] Hecobian A.; Liu Z.; Hennigan C. J.; Huey L. G.; Jimenez J. L.; Cubison M. J.; Vay S.; Diskin G. S.; Sachse G. W.; Wisthaler A.; Mikoviny T.; Weinheimer A. J.; Liao J.; Knapp D. J.; Wennberg P. O.; Kürten A.; Crounse J. D.; Clair J. S.; Wang Y.; Weber R. J. Comparison of chemical characteristics of 495 biomass burning plumes intercepted by the NASA DC-8 aircraft during the ARCTAS/CARB-2008 field campaign. Atmos. Chem. Phys. 2011, 11 (24), 13325–13337. 10.5194/acp-11-13325-2011.

[ref99] Pandit S.; Grassian V. H. Gas-Phase Nitrous Acid (HONO) Is Controlled by Surface Interactions of Adsorbed Nitrite (NO2−) on Common Indoor Material Surfaces. Environ. Sci. Technol. 2022, 56 (17), 12045–12054. 10.1021/acs.est.2c02042. 36001734 PMC9454260

[ref100] Zhang Q.; Wang Y.; Liu M.; Zheng M.; Yuan L.; Liu J.; Tao S.; Wang X. Wintertime Formation of Large Sulfate Particles in China and Implications for Human Health. Environ. Sci. Technol. 2023, 57, 20010 10.1021/acs.est.3c05645. 37909663 PMC10702544

[ref101] Zheng M.; Wang Y.; Bao J.; Yuan L.; Zheng H.; Yan Y.; Liu D.; Xie M.; Kong S. Initial Cost Barrier of Ammonia Control in Central China. Geophys. Res. Lett. 2019, 46 (23), 14175–14184. 10.1029/2019GL084351.

[ref102] Zhang W.; Tong S.; Jia C.; Wang L.; Liu B.; Tang G.; Ji D.; Hu B.; Liu Z.; Li W.; Wang Z.; Liu Y.; Wang Y.; Ge M. Different HONO Sources for Three Layers at the Urban Area of Beijing. Environ. Sci. Technol. 2020, 54 (20), 12870–12880. 10.1021/acs.est.0c02146. 32924447

[ref103] Jiang Y.; Xue L.; Shen H.; Dong C.; Xiao Z.; Wang W. Dominant Processes of HONO Derived from Multiple Field Observations in Contrasting Environments. Environmental Science & Technology Letters 2022, 9 (4), 258–264. 10.1021/acs.estlett.2c00004.

[ref104] Ye C.; Zhou X.; Pu D.; Stutz J.; Festa J.; Spolaor M.; Tsai C.; Cantrell C.; Mauldin R. L. 3rd; Campos T.; Weinheimer A.; Hornbrook R. S.; Apel E. C.; Guenther A.; Kaser L.; Yuan B.; Karl T.; Haggerty J.; Hall S.; Ullmann K.; Smith J. N.; Ortega J.; Knote C. Rapid cycling of reactive nitrogen in the marine boundary layer. Nature 2016, 532 (7600), 489–491. 10.1038/nature17195. 27064904

[ref105] Ramazan K. A.; Wingen L. M.; Miller Y.; Chaban G. M.; Gerber R. B.; Xantheas S. S.; Finlayson-Pitts B. J. New Experimental and Theoretical Approach to the Heterogeneous Hydrolysis of NO2: Key Role of Molecular Nitric Acid and Its Complexes. J. Phys. Chem. A 2006, 110 (21), 6886–6897. 10.1021/jp056426n. 16722704

[ref106] Zhou X.; Gao H.; He Y.; Huang G.; Bertman S. B.; Civerolo K.; Schwab J. Nitric acid photolysis on surfaces in low-NOx environments: Significant atmospheric implications. Geophys. Res. Lett. 2003, 30 (23), 2217 10.1029/2003GL018620.

[ref107] Hauglustaine D. A.; Ridley B. A.; Solomon S.; Hess P. G.; Madronich S. HNO3/NOx ratio in the remote troposphere During MLOPEX 2: Evidence for nitric acid reduction on carbonaceous aerosols?. Geophys. Res. Lett. 1996, 23 (19), 2609–2612. 10.1029/96GL02474.

[ref108] Liu Z.; Wang Y.; Gu D.; Zhao C.; Huey L. G.; Stickel R.; Liao J.; Shao M.; Zhu T.; Zeng L.; Amoroso A.; Costabile F.; Chang C. C.; Liu S. C. Summertime photochemistry during CAREBeijing-2007: ROx budgets and O3 formation. Atmospheric Chemistry and Physics 2012, 12 (16), 7737–7752. 10.5194/acp-12-7737-2012.

[ref109] Dyson J. E.; Boustead G. A.; Fleming L. T.; Blitz M.; Stone D.; Arnold S. R.; Whalley L. K.; Heard D. E. Production of HONO from NO2 uptake on illuminated TiO2 aerosol particles and following the illumination of mixed TiO2/ammonium nitrate particles. Atmos. Chem. Phys. 2021, 21 (7), 5755–5775. 10.5194/acp-21-5755-2021.

[ref110] Reed C.; Evans M. J.; Crilley L. R.; Bloss W. J.; Sherwen T.; Read K. A.; Lee J. D.; Carpenter L. J. Evidence for renoxification in the tropical marine boundary layer. Atmos. Chem. Phys. 2017, 17 (6), 4081–4092. 10.5194/acp-17-4081-2017.

[ref111] Kasibhatla P.; Sherwen T.; Evans M. J.; Carpenter L. J.; Reed C.; Alexander B.; Chen Q.; Sulprizio M. P.; Lee J. D.; Read K. A.; Bloss W.; Crilley L. R.; Keene W. C.; Pszenny A. A. P.; Hodzic A. Global impact of nitrate photolysis in sea-salt aerosol on NOx, OH, and O3 in the marine boundary layer. Atmospheric Chemistry and Physics 2018, 18 (15), 11185–11203. 10.5194/acp-18-11185-2018.

[ref112] Ha P. T. M.; Kanaya Y.; Taketani F.; Andrés Hernández M. D.; Schreiner B.; Pfeilsticker K.; Sudo K. Implementation of HONO into the chemistry–climate model CHASER (V4.0): roles in tropospheric chemistry. Geosci. Model Dev. 2023, 16 (3), 927–960. 10.5194/gmd-16-927-2023.

[ref113] Crilley L. R.; Kramer L. J.; Pope F. D.; Reed C.; Lee J. D.; Carpenter L. J.; Hollis L. D. J.; Ball S. M.; Bloss W. J. Is the ocean surface a source of nitrous acid (HONO) in the marine boundary layer?. Atmos. Chem. Phys. 2021, 21 (24), 18213–18225. 10.5194/acp-21-18213-2021.

[ref114] Wang P.; Chen Y.; Hu J.; Zhang H.; Ying Q. Attribution of Tropospheric Ozone to NOx and VOC Emissions: Considering Ozone Formation in the Transition Regime. Environ. Sci. Technol. 2019, 53 (3), 1404–1412. 10.1021/acs.est.8b05981. 30582806

[ref115] Ye C.; Zhou X.; Zhang Y.; Wang Y.; Wang J.; Zhang C.; Woodward-Massey R.; Cantrell C.; Mauldin R. L.; Campos T.; Hornbrook R. S.; Ortega J.; Apel E. C.; Haggerty J.; Hall S.; Ullmann K.; Weinheimer A.; Stutz J.; Karl T.; Smith J. N.; Guenther A.; Song S. Synthesizing evidence for the external cycling of NOx in high- to low-NOx atmospheres. Nat. Commun. 2023, 14 (1), 7995 10.1038/s41467-023-43866-z. 38042847 PMC10693570

[ref116] Tang Y.; An J.; Wang F.; Li Y.; Qu Y.; Chen Y.; Lin J. Impacts of an unknown daytime HONO source on the mixing ratio and budget of HONO, and hydroxyl, hydroperoxyl, and organic peroxy radicals, in the coastal regions of China. Atmos. Chem. Phys. 2015, 15 (16), 9381–9398. 10.5194/acp-15-9381-2015.

[ref117] Wang W.; Parrish D. D.; Wang S.; Bao F.; Ni R.; Li X.; Yang S.; Wang H.; Cheng Y.; Su H. Long-term trend of ozone pollution in China during 2014–2020: distinct seasonal and spatial characteristics and ozone sensitivity. Atmos. Chem. Phys. 2022, 22 (13), 8935–8949. 10.5194/acp-22-8935-2022.

[ref118] Yin H.; Lu X.; Sun Y.; Li K.; Gao M.; Zheng B.; Liu C. Unprecedented decline in summertime surface ozone over eastern China in 2020 comparably attributable to anthropogenic emission reductions and meteorology. Environmental Research Letters 2021, 16 (12), 124069 10.1088/1748-9326/ac3e22.

[ref119] Xu J.; Huang X.; Wang N.; Li Y.; Ding A. Understanding ozone pollution in the Yangtze River Delta of eastern China from the perspective of diurnal cycles. Science of The Total Environment 2021, 752, 141928 10.1016/j.scitotenv.2020.141928. 33207508 PMC7443166

[ref120] Li K.; Jacob D. J.; Shen L.; Lu X.; De Smedt I.; Liao H. Increases in surface ozone pollution in China from 2013 to 2019: anthropogenic and meteorological influences. Atmos. Chem. Phys. 2020, 20 (19), 11423–11433. 10.5194/acp-20-11423-2020.

