# Supplementary material for: Observation-Based Diagnostics of Reactive Nitrogen Recycling through HONO Heterogenous Production: Divergent Implications for Ozone Production and Emission Control
Source: Environ Sci Technol. 2024 Jun 17;58(26):11554–67. doi: 10.1021/acs.est.3c07967 (PMC11223480; doi:10.1021/acs.est.3c07967)
Supplement: Supplementary file 1 — es3c07967_si_001.pdf [file es3c07967_si_001.pdf]

Supporting Information

Observation-Based Diagnostics of Reactive Nitrogen Recycling through HONO Heterogenous Production: Divergent Implications for Ozone Production and Emission Control

Kezhen Chong[1], Yuhang Wang[1*], Mingming Zheng[2], Hang Qu[1], Ruixiong Zhang[1], Young Ro Lee[1], Yi Ji[1], Lewis Gregory Huey[1], Hua Fang[3, a], Wei Song[3], Zheng Fang[3, b], Cheng Liu[4], Yang Gao[5], Jianhui Tang[6], and Xinming Wang[3*]

[1] Georgia Institute of Technology, School of Earth and Atmospheric Sciences, Atlanta, GA 30332, United States

[2] Wuhan Polytechnic University, School of Chemical and Environmental Engineering, Wuhan 430024, China

[3] Guangzhou Institute of Geochemistry, Chinese Academy of Sciences, Guangzhou 510640, China

[4] University of Science and Technology of China, Hefei 230026, China

[5] Ocean University of China, Key Laboratory of Marine Environment and Ecology, Ministry of Education of China, Qingdao 266100, China

[6] Yantai Institute of Coast Zone Research, CAS, Yantai 264003, China

[a] now at: School of Ecology and Environment, Anhui Normal University, Wuhu 241000, China

[b] now at: Department of Earth and Planetary Sciences, Weizmann Institute of Science, Rehovot 76100, Israel

Number of pages: 20

Number of supporting texts: 2

Number of supporting figures: 15

Number of supporting tables: 6

Text S1. Descriptions of observations and data preparations

Text S2. HONO source parameterizations

Table S1. Instruments used during the OPECE campaign

Table S2. Correlation test results for biomass burning analysis

Table S3. Correlation test results for aerosol acidity effect analysis

Table S4. Statistics of correlation tests of pHONO against the production terms

Table S5. Comparisons of HONO source parameterizations to previous studies

Table S6. Comparisons of $HONO/NO_2$ and $HONO/pNO_3$ of different studies

Figure S1. Radius ratio of ambient to dry aerosols as a function of RH

Figure S2. Comparisons of AMS and $PM_{2.5}$ filter measurements of aerosol inorganic ions

Figure S3. Averaged diurnal profile and standard deviation (denoted by the vertical bars) of the mixing layer height diagnosed from K_{zz} during the study period.

Figure S4. Averaged noontime mixing ratios of VOCs during the study period

Figure S5. Comparisons of simulated HONO with photo-enhanced ground source with the observed HONO.

Figure S6. Averaged diurnal profile of the pNO_3 partitioning ratio during the study period.

Figure S7. Time series for O_3, NO_x, HONO, pNO_3, and S_A from 23 March to 22 April 2018

Figure S8. Mean diurnal profiles for observed O_3, NO_2, HONO, pNO_3, S_A, and NO

Figure S9. Correlations of CH_3CN with NO_2, pNO_3, S_A, and organic matters

Figure S10. Box plots for HONO, HONO/NO_2, and HONO/pNO_3 when $CH_3CN < 0.1$, $CH_3CN > 0.1$ and for the fire event on 31 Mar 2018

Figure S11. Scatter plots of nighttime HONO, HONO/NO_2, and HONO/pNO_3 against CH_3CN and aerosol pH

Figure S12. Frequency histogram for observation data points of different levels of CH_3CN

Figure S13. O_3 production efficiency as a function of initial NO_x concentrations

Figure S14. Annual mean MDA8 O_3 for eastern China from 2014 to 2022

Figure S15. Annual mean noontime NO_2 for eastern China from 2014 to 2022

Text S1. Descriptions of observations and data preparations

Calculation of ambient aerosol surface area

Aerosol size distributions were measured by the Scanning Mobility Particle Sizer (SMPS). To convert dry surface areas S_A^{dry} to ambient surface area (S_A) considering the hygroscopic growth, we apply the parameterization (Eq. S1) by Lewis[1]

$$\frac{r}{r_{dry}} = a\left(b + \frac{1}{1-h}\right)^{\frac{1}{3}} \qquad \text{(Eq S1)}$$

where r is the ambient aerosol radius, r_{dry} is the dry aerosol radius, h is relative humidity (RH), and a = 0.78 and b=1.90 are two empirical parameters chosen accordingly with the assumption of aerosol composition to be ammonium sulfate. The squared radius ratio is then used as surface area ratio to convert dry surface area to ambient wet surface area (Eq. S2).

$$S_A = S_A^{dry} \times \left(\frac{r}{r_{dry}}\right)^2 \qquad \text{(Eq S2)}$$

The calculated S_A at the surface is scaled by the vertical profile measured by Multi-Axis Differential Optical Absorption Spectroscopy (MAX-DOAS) to get S_A above the surface.

Data preparation for particulate nitrate (pNO_3) measurement

Particulate inorganic ions are measured at a 1-minute time step by the aerosol mass spectrometer (AMS) and daily by $PM_{2.5}$ filter samples. A comparison between mass concentrations of particulate nitrate (pNO_3), sulfate (SO_4^{2-}), ammonium (NH_4^+), and chloride (Cl^-) provided by AMS and $PM_{2.5}$ filter analysis shows a lower bias of AMS data for pNO_3, SO_4^{2-}, and Cl^-, except that filter-based NH_4^+ is lower than that measured by AMS, probably due to sampling artifacts[2-4]. In our study, we combine data from these two types of measurements as observed pNO_3. We first derive the hourly profile for each day from AMS data and then scale the hourly profile by the daily filter-based data.

Aerosol pH gas-particulate and partitioning calculations

The thermodynamic model ISOROPPIA-II (https://www.epfl.ch/labs/lapi/software/isorropia/)[5, 6] was used to compute aerosol pH and partition of soluble species between particulate and gaseous phases. The forward mode with the inputs of total species concentrations (i.e., gas + particle) and meteorological parameters (relative humidity, temperature) gives more accurate results than the reverse mode.[6-9] Due to a lack of observed NH3 and HNO3 data, we iterated ISORROPIA-II adopting the predicted NH3 and HNO3 to obtain total ammonia (NH3, NH4+) and nitrate (HNO3+, NO3-) concentrations as inputs for the next iteration until NH3 and HNO3 predictions converge.[10]

Text S2. HONO source parameterizations

Heterogeneous conversion of NO_2 on ground surfaces

We adopt the parameterization by Liu et al.[11] to simulate HONO formation from the ground conversion of NO_2. The production of HONO is parameterized as a subsequent release following NO_2 dry deposition on the ground into the surface layer. The parameterization is

$$\text{prod (HONO)} = f \times [NO_2] \times V_d / H \qquad \text{(Eq S3)}$$

where f is the yield of HONO from NO_2 reaching the surface; H is the height of the first model layer, and V_d is the dry deposition velocity of NO_2. Since the photoactive mechanisms have negligible impact at night, we estimate the yield coefficient (f) for HONO from deposited NO_2 so that simulated nocturnal HONO can match observed concentrations.

Photosensitized conversion from NO_2 on aerosols

We parameterize photosensitized NO_2 conversion on aerosol surfaces as a first order reaction:

$$\text{prod (HONO)} = k_a \times [NO_2],\ k_a = S_A\left[\frac{R_p}{D_g} + \frac{4}{\gamma\omega}\right]^{-1} \quad \text{(Eq S4)}$$

where S_A is the aerosol surface area, R_p ~ 0.1μm is the aerosol radius; D_g is the NO_2 molecular diffusion coefficient; ω is the mean molecular speed for NO_2 and γ is the aerosol uptake coefficient. Recent studies show that sunlight can catalyze this process. To represent this photo-enhanced characteristic, we consider a first-order enhancement from short wave radiation (SWR) to γ

$$\gamma = \text{SWR} \times \gamma' \quad \text{(Eq S5)}$$

where SWR is simulated by WRF and scaled by observed jNO_2, and γ' is a constant.

Conversion from pNO_3

Ye et al.[12] showed that the photolysis rate of pNO_3 has a median value of 8.3×10^{-5} s^{-1} and it is usually computed by scaling the photolysis rate of gas-phase nitric acid ($jHNO_3$) to get the photolysis rate of pNO_3 ($jpNO_3$):

$$\text{prod (HONO)} = jpNO3 \times [pNO_3] \quad \text{(Eq S6)}$$

$$jpNO_3 = EF \times jHNO_3 \quad \text{(Eq S7)}$$

where EF is the enhancement factor. As Andersen et al.[13] have pointed out, EF can be dependent on pNO_3, through the Langmuir function. Therefore, in addition to selecting a constant EF to reproduce the observed HONO as in case S2-1, we also fitted this relationship of EF on pNO_3 as described in the following equation, where K_L = 0.19 $nmol^{-1}m^3$ is the Langmuir equilibrium constant of nitrate ion, and a is the coefficient to be fitted:

$$EF = \frac{K_L \times a}{1 + K_L \times [pNO_3]} \quad \text{(Eq S8)}$$

Conversion from HNO_3

Song et al.[14] showed that nitric acid (HNO_3) from photooxidation of NO_2 can be converted into HONO with a yield coefficient (Y_{HONO}) of 53%. In our study, we followed the same parameterization as described in their study to incorporate the HONO source from HNO_3.

$$\text{prod (HONO)} = Y_{HONO} \times P(HNO_3 \text{ from } NO_2\text{+OH}) \quad \text{(Eq S9)}$$

Tables

Table S1. Instruments used during the OPECE campaign

**Parameters**	**Instruments**	**Temporal resolution**
HONO	LOPAP	1 min
NO, NO_2	Thermo 42i	1 min
O_3	Thermo 49i	1 min
**CO**	Thermo 48i	1 min
**VOCs**	GCFID or PTR-ToF-MS	60min or 1min
Aerosol number size distribution	Scanning Mobility Particle Sizer (SMPS)	5 min
CH_3CN	PTR-ToF-MS	1 min
**jNO_2**	Actinic flux	1 min
Aerosol chemical composition	Sample filter analysis	24 hour
	Aerosol Mass Spectrometer (AMS)	1 min

Table S2. Correlation coefficient (r) and p values for the correlation analysis of CH_3CN with HONO, HONO/NO_2, HONO/pNO_3, pNO_3, and NO_2.

Correlation with CH_3CN	HONO		HONO/NO_2		HONO/pNO_3		pNO_3		NO_2	
	r	p	r	p	r	p	r	p	r	p
Daytime	**0.60**	**9×10^{-10}**	**-0.30**	**0.005**	**-0.32**	**0.003**	**0.49**	**4×10^{-9}**	**0.72**	**1×10^{-28}**
Nighttime	**0.41**	**5×10^{-4}**	-0.14	0.250	0.19	0.115	-0.16	0.10	**0.4**	**3×10^{-6}**

Table S3. Correlation coefficients (r) and p values for the correlation analysis of pH with HONO, HONO/NO_2, HONO/pNO_3, SWR, and S_A.

Correlation with pH	HONO		$HONO/NO_2$		$HONO/pNO_3$		SWR		S_A	
	r	p	r	p	r	p	r	p	r	p
Daytime	-0.10	0.40	-0.03	0.8	-0.07	0.67	0.05	0.6	-0.15	0.14
Nighttime	**-0.27**	**8×10^{-3}**	**-0.46**	**6×10^{-6}**	**0.57**	**1×10^{-5}**	\	\	**-0.31**	**0.002**

Table S4. Statistics of correlation tests of pHONO with the production terms.

Source	Production Terms	R	Sample Size
Photosensitized NO_2 conversion on aerosols	SWR	0.43	84
	NO_2	0.83	
	$SWR*NO_2*SFCA$	0.9	
Photolysis of pNO_3	$jHNO_3$	0.53	57
	pNO_3	0.83	
	pNO_3*jHNO_3	0.87	
HNO_3 conversion	$PHNO_3$	0.9	93

Table S5. Comparisons of HONO source parameterizations to previous studies

**Location**	**Time**	**γ**		**$jpNO_3$ [s^{-1}]**		**Y_{HONO}**	**Reference**
Laboratory		3×10^{-4}					Colussi et al.[15]
Laboratory						0.53	Song et al.[14]
Laboratory		1×10^{-5}					Han et al.[16]
Laboratory		2×10^{-5}					Stemmler et al.[17]
Beijing, China	Aug 2007	$1\times10^{-4}\times k_{SWR}{}^{I}$					Liu et al.[11]
Houston, TX, US	Apr-May 2009	$6\times10^{-5}\times k_{jNO_2}{}^{II}$					Wong et al.[18]
Dallas, TX, US	Jun 2011	$8.5\times10^{-4}\times k_{jNO_2}$					Gall et al.[19]
Hong Kong, China	Aug 2011	$5\times10^{-5}\times k_{SWR}$					Zhang et al.[20]
Laboratory	Multiple years			$(8 – 700)\times jHNO_3$			Ye et al.[12]
Wangdu, Hebei, China	Jun - Jul 2014	$1\times10^{-3}\times k_{jNO_2}$	and	1.3×10^{-4}			Liu et al.[21]

Laboratory	Apr-Dec 2016			8.2×10^{-5}			Bao et al.[22]
Nanjing, Jiangsu, China	May-Jun 2016	$2 \times 10^{-5} \times k_{jNO_2}$	and	$208 \times jHNO_3$			Ge et al.[23]
Yellow Sea	Summer 2016			$30 \times jHNO_3$			Romer et al.[24]
Optimal γ	Multiple cases	$5 \times 10^{-4} \times k_{SWR}$					Lu et al.[25]
Changzhou, Jiangsu, China	Apr 2017	$2 \times 10^{-5} \times k_{jNO_2}$	and	$119 \times jHNO_3$			Shi et al.[26]
Beijing, China	Apr-May 2018	$1.1 \times 10^{-4} \times k_{SWR}$	and	$119 \times jHNO_3$			Zhang et al.[27]
Beijing, China	May-Jul 2018	$1 \times 10^{-4} \times k_{jNO_2}$	and	$119 \times jHNO_3$			Liu et al.[28]
Beijing, China	Nov 2018 - Jan 2019	$6 \times 10^{-5} \times k_{jNO_2}$	and	$119 \times jHNO_3$			Liu et al.[28]
THMAO site, Bermuda	Spring & late summer 2019			$29 \times jHNO_3$			Zhu et al.[29]
Dongying, Shandong, China	Mar-Apr 2018	$5 \times 10^{-4} \times k_{SWR}$	or	$EF \times jHNO_3$, where EF=80 or $f(pNO_3)$ with resulting EF ranges from 20 to 3600 with a median of 106	or	0.45	This study

[I] $k_{SWR} = \frac{SWR}{1000}$

[II] $k_{jNO_2} = \frac{jNO_2}{jNO_2 \text{ at noon}}$

Table S6. Comparisons of $HONO/NO_2$ and $HONO/pNO_3$ of different studies

Location	Time	$\mathbf{HONO/NO_2}$	$\mathbf{HONO/pNO_3}$	Reference
Beijing, China	Aug 2007	9.5%		Liu et al.[11]
Houston, TX, US	Apr - May 2009	2.5%		Wong et al.[18]
Dallas, TX, US	Jun 2011	1%		Gall et al.[19]
Hong Kong, China	Aug 2011	2.6%		Zhang et al.[20]
Wangdu, Hebei, China	Jun - Jul 2014	5%	14%	Liu et al.[21]
Cape Verde GAW station	2014 - 2015	13%	0.9%	Reed et al.[30]
Nanjing, Jiangsu, China	May - Jun 2016	5%	12%	Ge et al.[23]
Changzhou, Jiangsu, China	Apr 2017	6.8%	41%	Shi et al.[26]
Beijing, China	Apr – May 2018	11%		Zhang et al.[27]
Beijing, China	May - Jul 2018	7%		Liu et al.[28]
Beijing, China	Nov 2018 - Jan 2019	5%		Liu et al.[28]
Dongying, Shandong, China	Mar - Apr 2018	4.3%	4.1%	This study

Figures

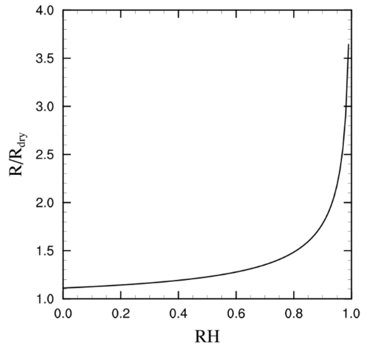

Figure S1. Radius ratio of ambient to dry aerosols as a function of RH.

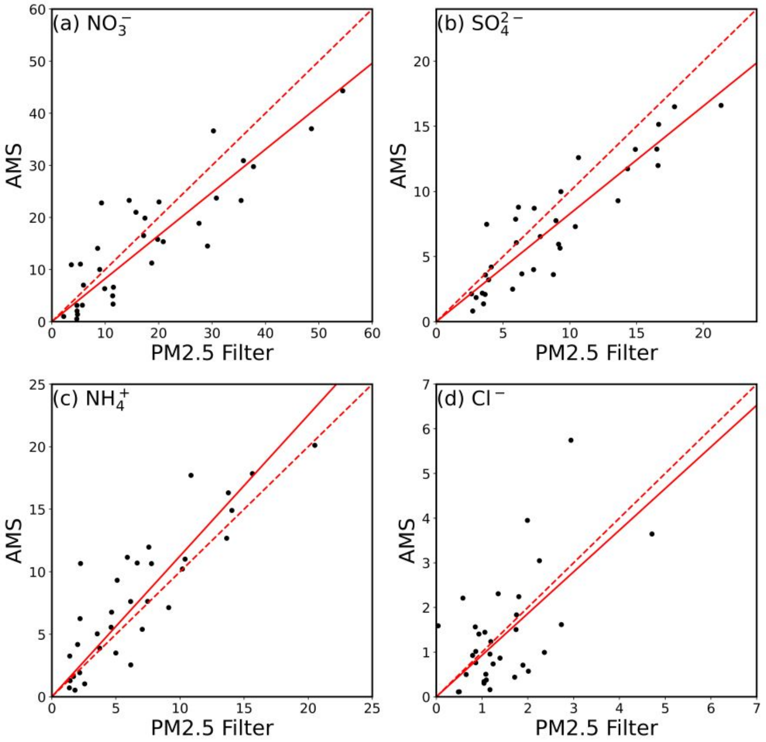

Figure S2. Comparisons of daily AMS and $PM_{2.5}$ filter measurements of aerosol inorganic ions: (a) pNO_3, (b) SO_4^{2-}, (c) NH_4^+, and (d) Cl^-. Red solid lines denote through-the-origin least-squares regressions and red dashed lines are 1:1 lines.

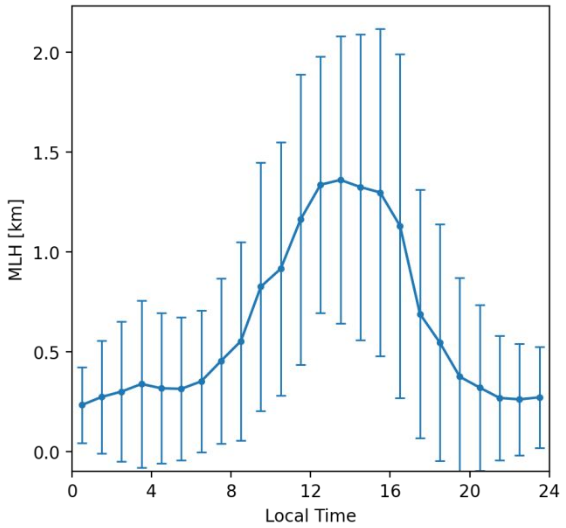

Figure S3. Averaged diurnal profile and standard deviation (denoted by the vertical bars) of the mixing layer height diagnosed from K_{zz} during the study period.

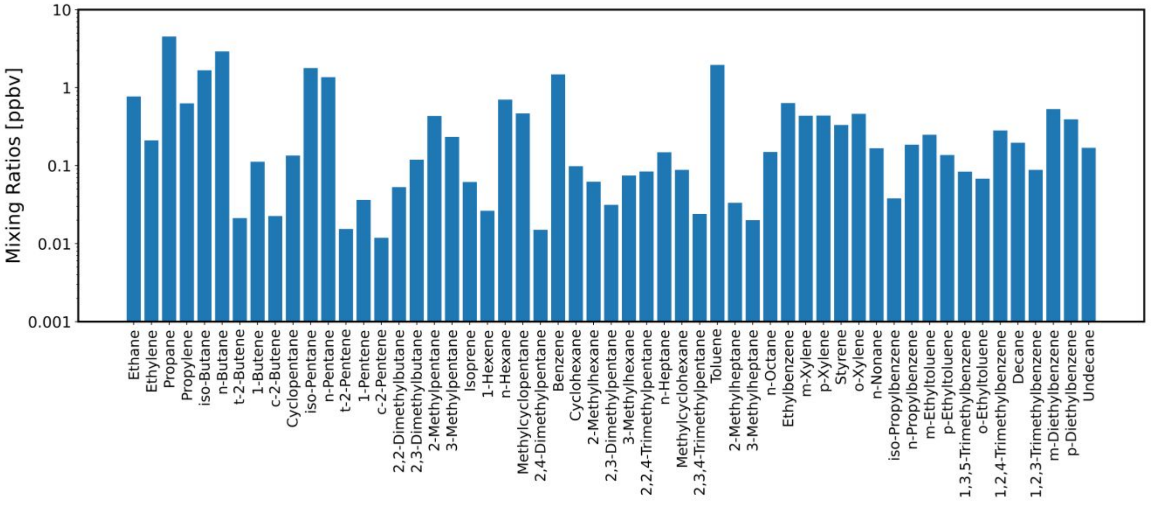

Figure S4. Averaged mixing ratios of VOCs between 10:00 to 15:00 local time (LT) during the study period.

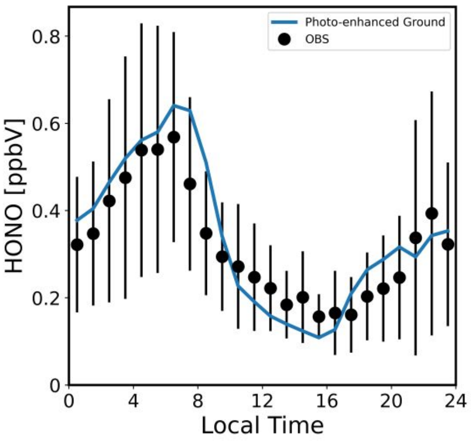

Figure S5. Comparisons of simulated HONO with photo-enhanced ground source with the observed HONO.

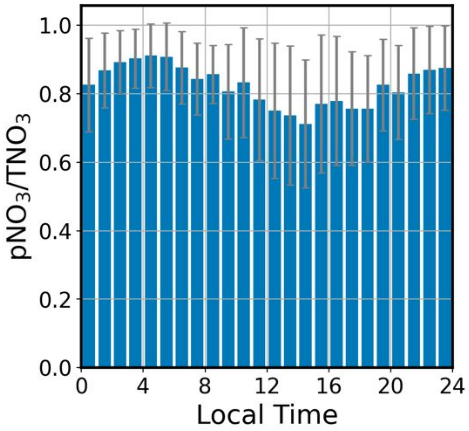

Figure S6. Averaged diurnal profile of the pNO_3 partitioning ratio (pNO_3/TNO_3, $TNO_3=pNO_3+HNO_3$) during the study period.

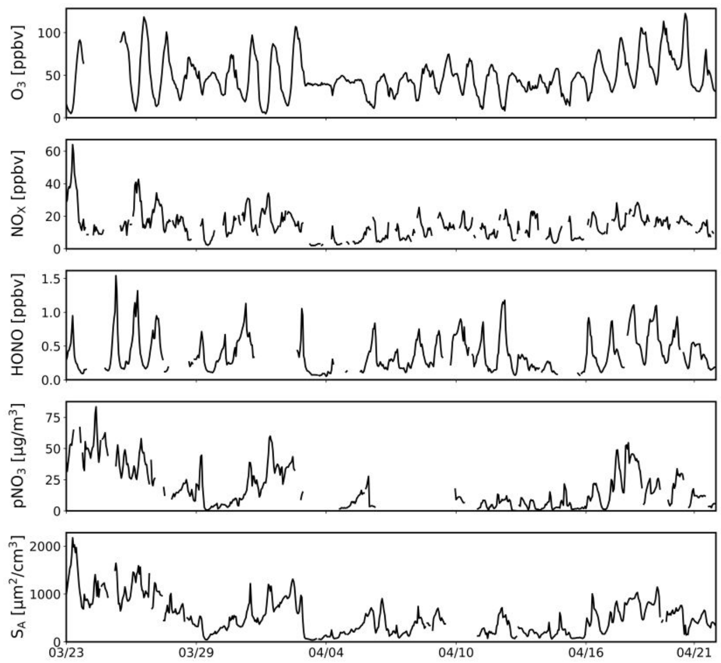

Figure S7. Time series for O_3, NO_x, HONO, pNO_3, and S_A from 23 March to 22 April 2018.

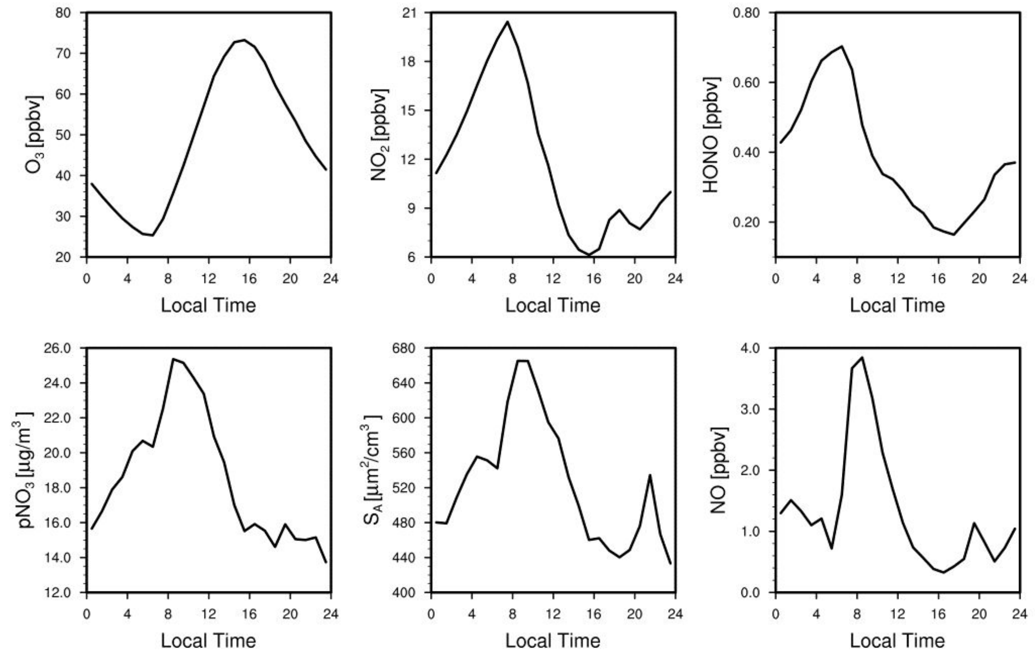

Figure S8. Mean diurnal profiles for observed O_3, NO_2, HONO, pNO_3, S_A, and NO.

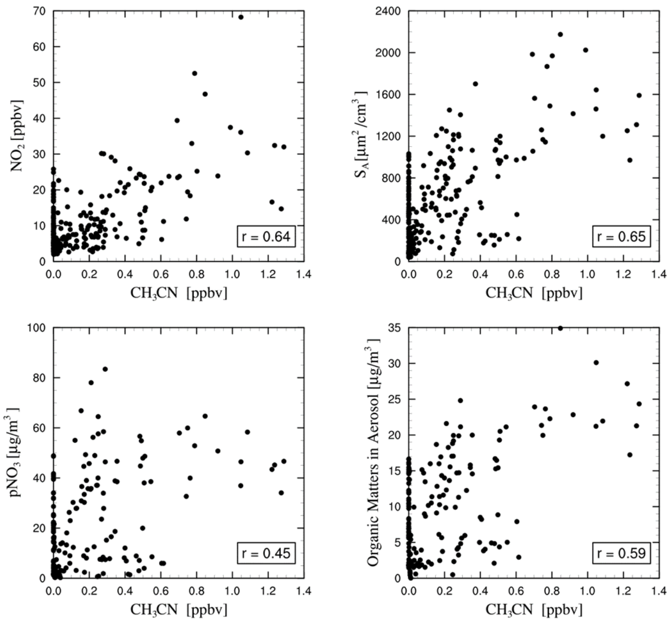

Figure S9. Correlations of CH_3CN with NO_2, pNO_3, S_A and organic matters in aerosol.

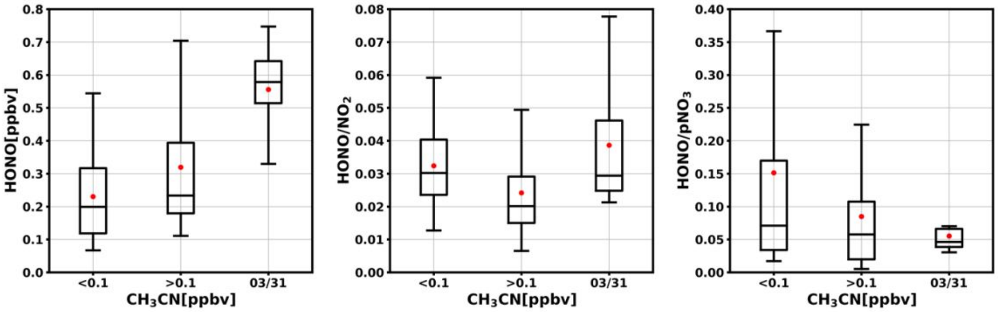

Figure S10. Box plots for HONO, HONO/NO_2, and HONO/pNO_3 when $CH_3CN < 0.1$, $CH_3CN > 0.1$ and for the fire event on 31 Mar 2018.

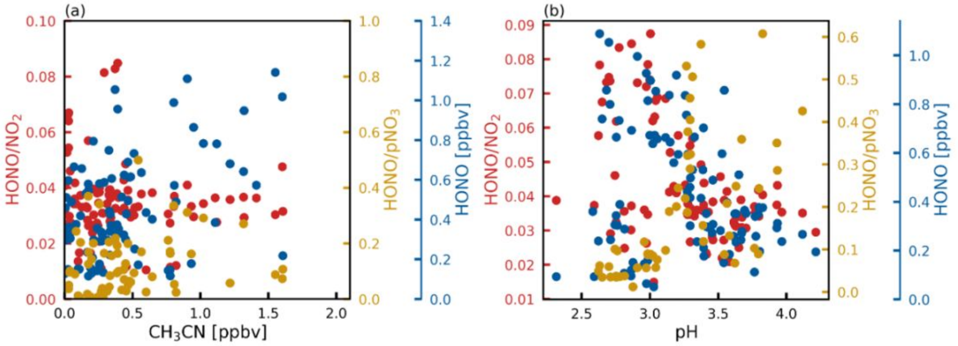

Figure S11. (a) Scatter plots of nighttime (20:00 – 05:00) HONO, HONO/NO_2, and HONO/pNO_3 as functions of CH_3CN. (b) Same as (a) but for aerosol pH.

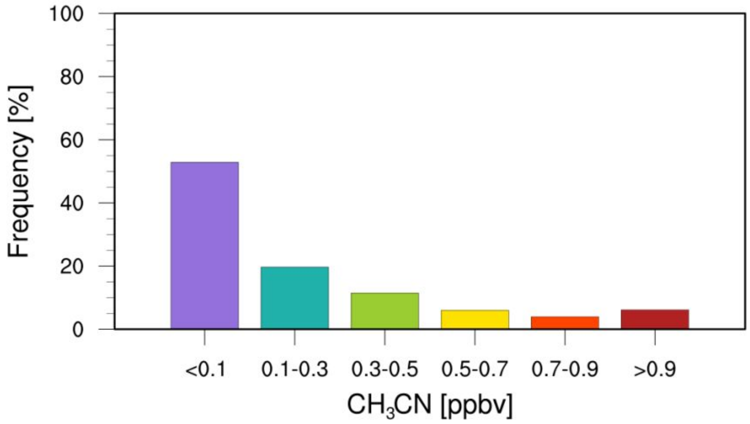

Figure S12. Frequency histogram for observation data points of different levels of CH_3CN.

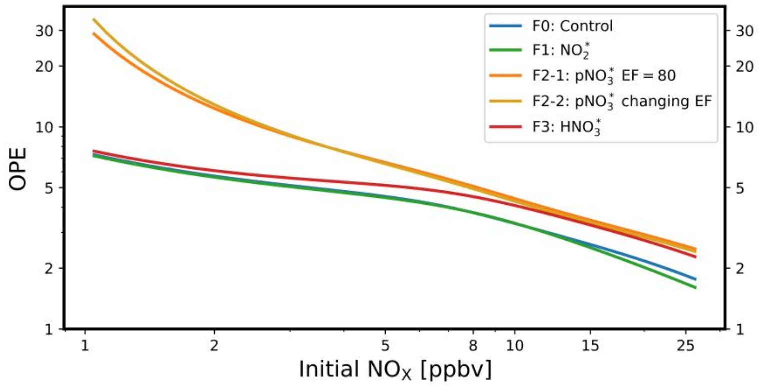

Figure S13. Ozone production efficiency (OPE) as a function of initial NO_x concentrations for cases F0 - F3.

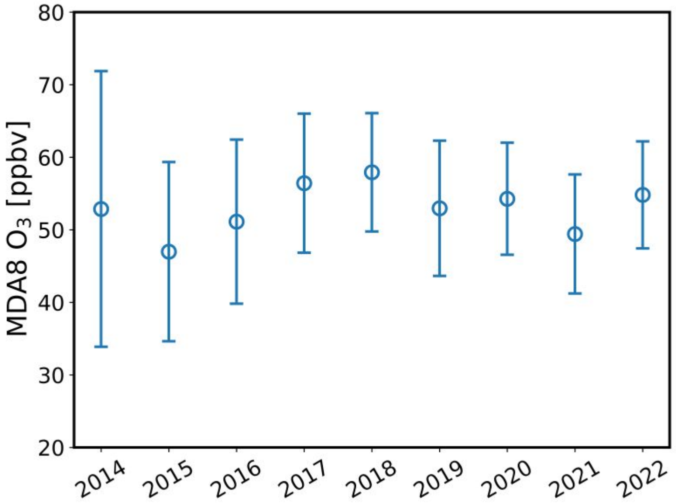

Figure S14. Annual mean maximum daily average 8-hr (MDA8) O_3 concentrations for eastern China from 2014 to 2022.

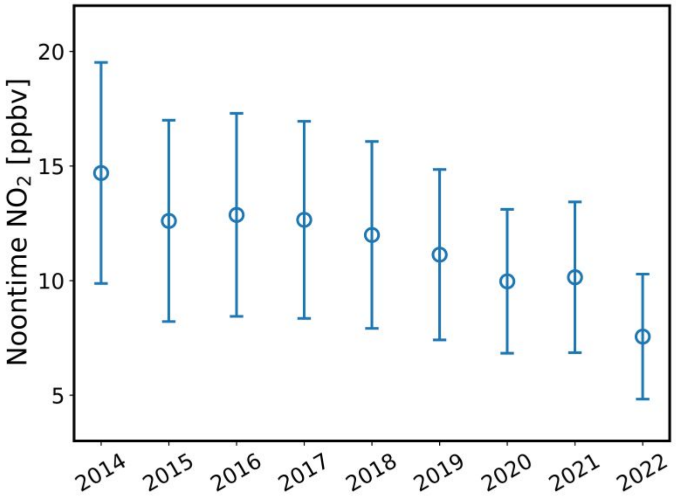

Figure S15. Annual mean noontime NO_2 concentrations for eastern China from 2014 to 2022.

References.

(1) Lewis, E. R. An examination of Köhler theory resulting in an accurate expression for the equilibrium radius ratio of a hygroscopic aerosol particle valid up to and including relative humidity 100%. *Journal of Geophysical Research* **2008**, *113* (D3). DOI: 10.1029/2007jd008590.
(2) Lipfert, F. W. Filter artifacts associated with particulate measurements: Recent evidence and effects on statistical relationships. *Atmospheric Environment* **1994**, *28* (20), 3233-3249. DOI: https://doi.org/10.1016/1352-2310(94)00167-J.
(3) Vecchi, R.; Valli, G.; Fermo, P.; D'Alessandro, A.; Piazzalunga, A.; Bernardoni, V. Organic and inorganic sampling artefacts assessment. *Atmospheric Environment* **2009**, *43* (10), 1713-1720. DOI: 10.1016/j.atmosenv.2008.12.016.
(4) Du, H.; Kong, L.; Cheng, T.; Chen, J.; Yang, X.; Zhang, R.; Han, Z.; Yan, Z.; Ma, Y. Insights into Ammonium Particle-to-Gas Conversion: Non-sulfate Ammonium Coupling with Nitrate and Chloride. *Aerosol and Air Quality Research* **2010**, *10* (6), 589-595. DOI: 10.4209/aaqr.2010.04.0034.
(5) Nenes, A.; Pandis, S. N.; Pilinis, C. ISORROPIA: A New Thermodynamic Equilibrium Model for Multiphase Multicomponent Inorganic Aerosols. *Aquatic Geochemistry* **1998**, *4* (1), 123-152. DOI: 10.1023/A:1009604003981.
(6) Fountoukis, C.; Nenes, A. ISORROPIA II: a computationally efficient thermodynamic equilibrium model for K+–Ca2+–Mg2+–NH+
4 –Na+–SO2− 4 –NO− 3 –Cl−–H2O aerosols. *Atmos. Chem. Phys.* **2007**, *7* (17), 4639-4659. DOI: 10.5194/acp-7-4639-2007.
(7) Guo, H.; Xu, L.; Bougiatioti, A.; Cerully, K. M.; Capps, S. L.; Hite Jr, J. R.; Carlton, A. G.; Lee, S. H.; Bergin, M. H.; Ng, N. L.; Nenes, A.; Weber, R. J. Fine-particle water and pH in the southeastern United States. *Atmos. Chem. Phys.* **2015**, *15* (9), 5211-5228. DOI: 10.5194/acp-15-5211-2015.
(8) Hennigan, C. J.; Izumi, J.; Sullivan, A. P.; Weber, R. J.; Nenes, A. A critical evaluation of proxy methods used to estimate the acidity of atmospheric particles. *Atmos. Chem. Phys.* **2015**, *15* (5), 2775-2790. DOI: 10.5194/acp-15-2775-2015.
(9) Weber, R. J.; Guo, H.; Russell, A. G.; Nenes, A. High aerosol acidity despite declining atmospheric sulfate concentrations over the past 15 years. *Nature Geoscience* **2016**, *9* (4), 282-285. DOI: 10.1038/ngeo2665.
(10) Guo, H.; Sullivan, A. P.; Campuzano-Jost, P.; Schroder, J. C.; Lopez-Hilfiker, F. D.; Dibb, J. E.; Jimenez, J. L.; Thornton, J. A.; Brown, S. S.; Nenes, A.; Weber, R. J. Fine particle pH and the partitioning of nitric acid during winter in the northeastern United States. *Journal of Geophysical Research: Atmospheres* **2016**, *121* (17), 10,355-310,376, https://doi.org/10.1002/2016JD025311. DOI: https://doi.org/10.1002/2016JD025311 (acccessed 2021/08/26).
(11) Liu, Z.; Wang, Y.; Costabile, F.; Amoroso, A.; Zhao, C.; Huey, L. G.; Stickel, R.; Liao, J.; Zhu, T. Evidence of aerosols as a media for rapid daytime HONO production over China. *Environ Sci Technol* **2014**, *48* (24), 14386-14391. DOI: 10.1021/es504163z.
(12) Ye, C.; Zhang, N.; Gao, H.; Zhou, X. Photolysis of Particulate Nitrate as a Source of HONO and NOx. *Environ Sci Technol* **2017**, *51* (12), 6849-6856. DOI: 10.1021/acs.est.7b00387.
(13) Andersen, S. T.; Carpenter, L. J.; Reed, C.; Lee, J. D.; Chance, R.; Sherwen, T.; Vaughan, A. R.; Stewart, J.; Edwards, P. M.; Bloss, W. J.; Sommariva, R.; Crilley, L. R.; Nott, G. J.; Neves, L.; Read, K.; Heard, D. E.; Seakins, P. W.; Whalley, L. K.; Boustead, G. A.; Fleming, L. T.; Stone, D.; Fomba, K. W.

Extensive field evidence for the release of HONO from the photolysis of nitrate aerosols. *Science Advances* **2023**, *9* (3), eadd6266. DOI: doi:10.1126/sciadv.add6266.
(14) Song, M.; Zhao, X.; Liu, P.; Mu, J.; He, G.; Zhang, C.; Tong, S.; Xue, C.; Zhao, X.; Ge, M.; Mu, Y. Atmospheric NOx oxidation as major sources for nitrous acid (HONO). *npj Climate and Atmospheric Science* **2023**, *6* (1), 30. DOI: 10.1038/s41612-023-00357-8.
(15) Colussi, A. J.; Enami, S.; Yabushita, A.; Hoffmann, M. R.; Liu, W. G.; Mishra, H.; Goddard, W. A., 3rd. Tropospheric aerosol as a reactive intermediate. *Faraday Discuss* **2013**, *165*, 407-420. DOI: 10.1039/c3fd00040k.
(16) Han, C.; Yang, W.; Wu, Q.; Yang, H.; Xue, X. Heterogeneous Photochemical Conversion of NO2 to HONO on the Humic Acid Surface under Simulated Sunlight. *Environmental Science & Technology* **2016**, *50* (10), 5017-5023. DOI: 10.1021/acs.est.5b05101.
(17) Stemmler, K.; Ammann, M.; Donders, C.; Kleffmann, J.; George, C. Photosensitized reduction of nitrogen dioxide on humic acid as a source of nitrous acid. *Nature* **2006**, *440* (7081), 195-198. DOI: 10.1038/nature04603.
(18) Wong, K. W.; Tsai, C.; Lefer, B.; Grossberg, N.; Stutz, J. Modeling of daytime HONO vertical gradients during SHARP 2009. *Atmospheric Chemistry and Physics* **2013**, *13* (7), 3587-3601. DOI: 10.5194/acp-13-3587-2013.
(19) Gall, E. T.; Griffin, R. J.; Steiner, A. L.; Dibb, J.; Scheuer, E.; Gong, L.; Rutter, A. P.; Cevik, B. K.; Kim, S.; Lefer, B.; Flynn, J. Evaluation of nitrous acid sources and sinks in urban outflow. *Atmospheric Environment* **2016**, *127*, 272-282. DOI: 10.1016/j.atmosenv.2015.12.044.
(20) Zhang, L.; Wang, T.; Zhang, Q.; Zheng, J.; Xu, Z.; Lv, M. Potential sources of nitrous acid (HONO) and their impacts on ozone: A WRF-Chem study in a polluted subtropical region. *Journal of Geophysical Research: Atmospheres* **2016**, *121* (7), 3645-3662. DOI: 10.1002/2015jd024468.
(21) Liu, Y.; Lu, K.; Li, X.; Dong, H.; Tan, Z.; Wang, H.; Zou, Q.; Wu, Y.; Zeng, L.; Hu, M.; Min, K. E.; Kecorius, S.; Wiedensohler, A.; Zhang, Y. A Comprehensive Model Test of the HONO Sources Constrained to Field Measurements at Rural North China Plain. *Environ Sci Technol* **2019**, *53* (7), 3517-3525. DOI: 10.1021/acs.est.8b06367.
(22) Bao, F.; Li, M.; Zhang, Y.; Chen, C.; Zhao, J. Photochemical Aging of Beijing Urban PM2.5: HONO Production. *Environ Sci Technol* **2018**, *52* (11), 6309-6316. DOI: 10.1021/acs.est.8b00538.
(23) Ge, Y.; Shi, X.; Ma, Y.; Zhang, W.; Ren, X.; Zheng, J.; Zhang, Y. Seasonality of nitrous acid near an industry zone in the Yangtze River Delta region of China: Formation mechanisms and contribution to the atmospheric oxidation capacity. *Atmospheric Environment* **2021**, *254*. DOI: 10.1016/j.atmosenv.2021.118420.
(24) Romer, P. S.; Wooldridge, P. J.; Crounse, J. D.; Kim, M. J.; Wennberg, P. O.; Dibb, J. E.; Scheuer, E.; Blake, D. R.; Meinardi, S.; Brosius, A. L.; Thames, A. B.; Miller, D. O.; Brune, W. H.; Hall, S. R.; Ryerson, T. B.; Cohen, R. C. Constraints on Aerosol Nitrate Photolysis as a Potential Source of HONO and NO x. *Environ Sci Technol* **2018**, *52* (23), 13738-13746. DOI: 10.1021/acs.est.8b03861.
(25) Lu, X.; Wang, Y.; Li, J.; Shen, L.; Fung, J. C. H. Evidence of heterogeneous HONO formation from aerosols and the regional photochemical impact of this HONO source. *Environmental Research Letters* **2018**, *13* (11). DOI: 10.1088/1748-9326/aae492.
(26) Shi, X.; Ge, Y.; Zheng, J.; Ma, Y.; Ren, X.; Zhang, Y. Budget of nitrous acid and its impacts on atmospheric oxidative capacity at an urban site in the central Yangtze River Delta region of China. *Atmospheric Environment* **2020**, *238*. DOI: 10.1016/j.atmosenv.2020.117725.
(27) Zhang, W.; Tong, S.; Jia, C.; Wang, L.; Liu, B.; Tang, G.; Ji, D.; Hu, B.; Liu, Z.; Li, W.; Wang, Z.; Liu, Y.; Wang, Y.; Ge, M. Different HONO Sources for Three Layers at the Urban Area of Beijing. *Environ Sci Technol* **2020**, *54* (20), 12870-12880. DOI: 10.1021/acs.est.0c02146.

(28) Liu, J.; Liu, Z.; Ma, Z.; Yang, S.; Yao, D.; Zhao, S.; Hu, B.; Tang, G.; Sun, J.; Cheng, M.; Xu, Z.; Wang, Y. Detailed budget analysis of HONO in Beijing, China: Implication on atmosphere oxidation capacity in polluted megacity. *Atmospheric Environment* **2021**, *244*. DOI: 10.1016/j.atmosenv.2020.117957.
(29) Zhu, Y.; Wang, Y.; Zhou, X.; Elshorbany, Y. F.; Ye, C.; Hayden, M.; Peters, A. J. An investigation into the chemistry of HONO in the marine boundary layer at Tudor Hill Marine Atmospheric Observatory in Bermuda. *Atmos. Chem. Phys.* **2022**, *22* (9), 6327-6346. DOI: 10.5194/acp-22-6327-2022.
(30) Reed, C.; Evans, M. J.; Crilley, L. R.; Bloss, W. J.; Sherwen, T.; Read, K. A.; Lee, J. D.; Carpenter, L. J. Evidence for renoxification in the tropical marine boundary layer. *Atmos. Chem. Phys.* **2017**, *17* (6), 4081-4092. DOI: 10.5194/acp-17-4081-2017.
